# Supplementary material for: Individuality and convergence of the infant gut microbiota during the first year of life
Source: Nat Commun. 2018 Jun 8;9:2233. doi: 10.1038/s41467-018-04641-7 (PMC5993781; doi:10.1038/s41467-018-04641-7)
Supplement: Supplementary file 1 — Supplementary Information [file 41467_2018_4641_MOESM1_ESM.pdf]

Individuality and convergence of the infant gut microbiota during the first year of life

de Muinck and Trosvik

Supplementary Figures 1-30. Supplementary Table 1.

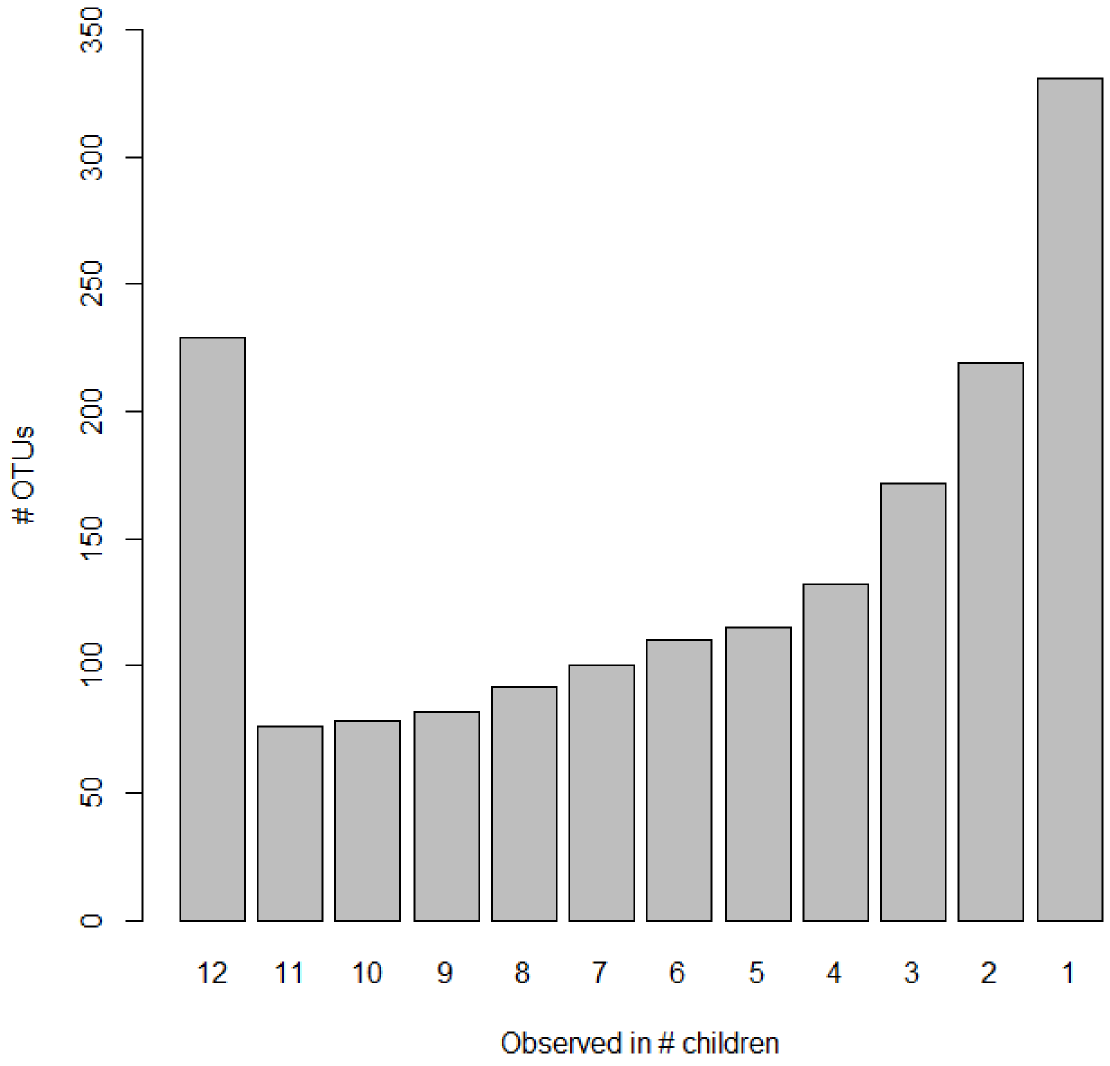

Supplementary Figure 1 | Distribution of Shared OTUs.
The bars indicate the number of OTUs shared (over the combined time series) among the number of infants indicated on the x-axis. 229 OTUs were observed in all 12 infants. 331 OTUs were unique to one infant.

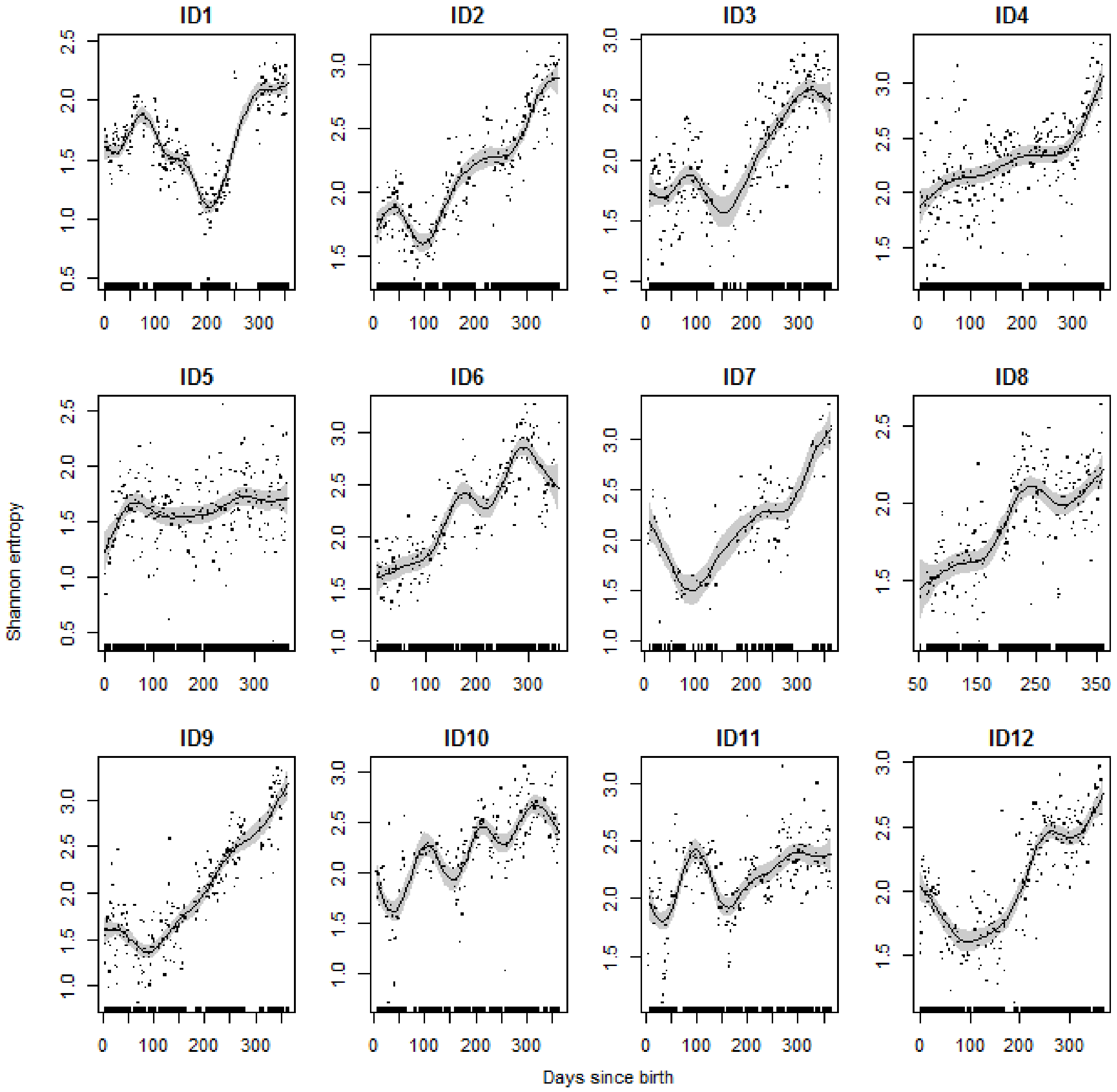

Supplementary Figure 2 | Diversity over time as measured by Shannon entropy. The fitted lines are generalized additive models using 9 degrees of freedom for estimating the smooth terms. Shaded areas represent 95% confidence limits. The rugs along the x-axes indicate days for which we had samples.

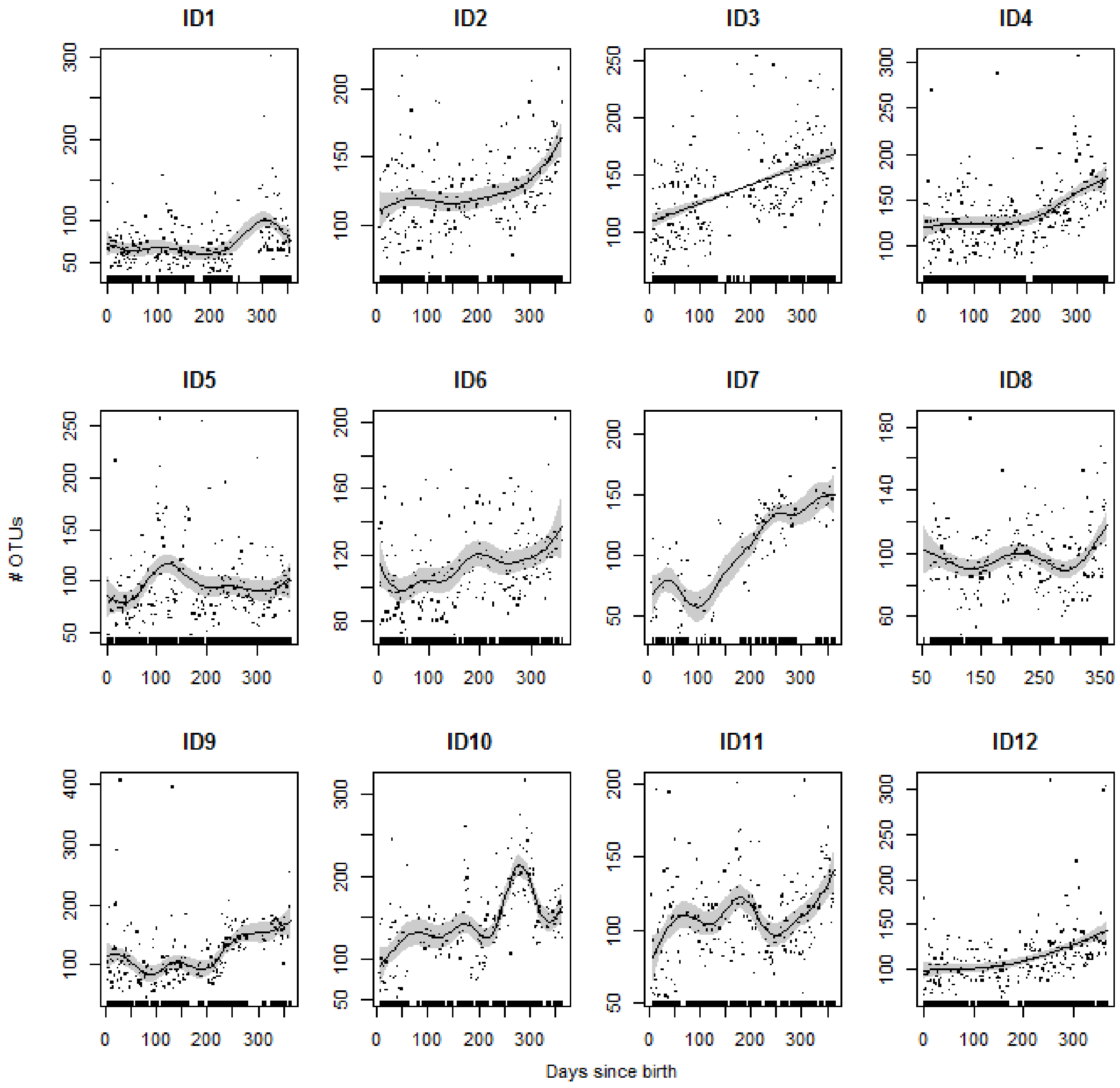

Supplementary Figure 3 | OTU richness in the 12 infants over the first year of life. The number of OTUs observed at each time point is indicated on the y-axes. The fitted lines are generalized additive models using 9 degrees of freedom for estimating the smooth terms. Shaded areas represent 95% confidence limits. The rugs along the x-axis indicate days for which we had samples.

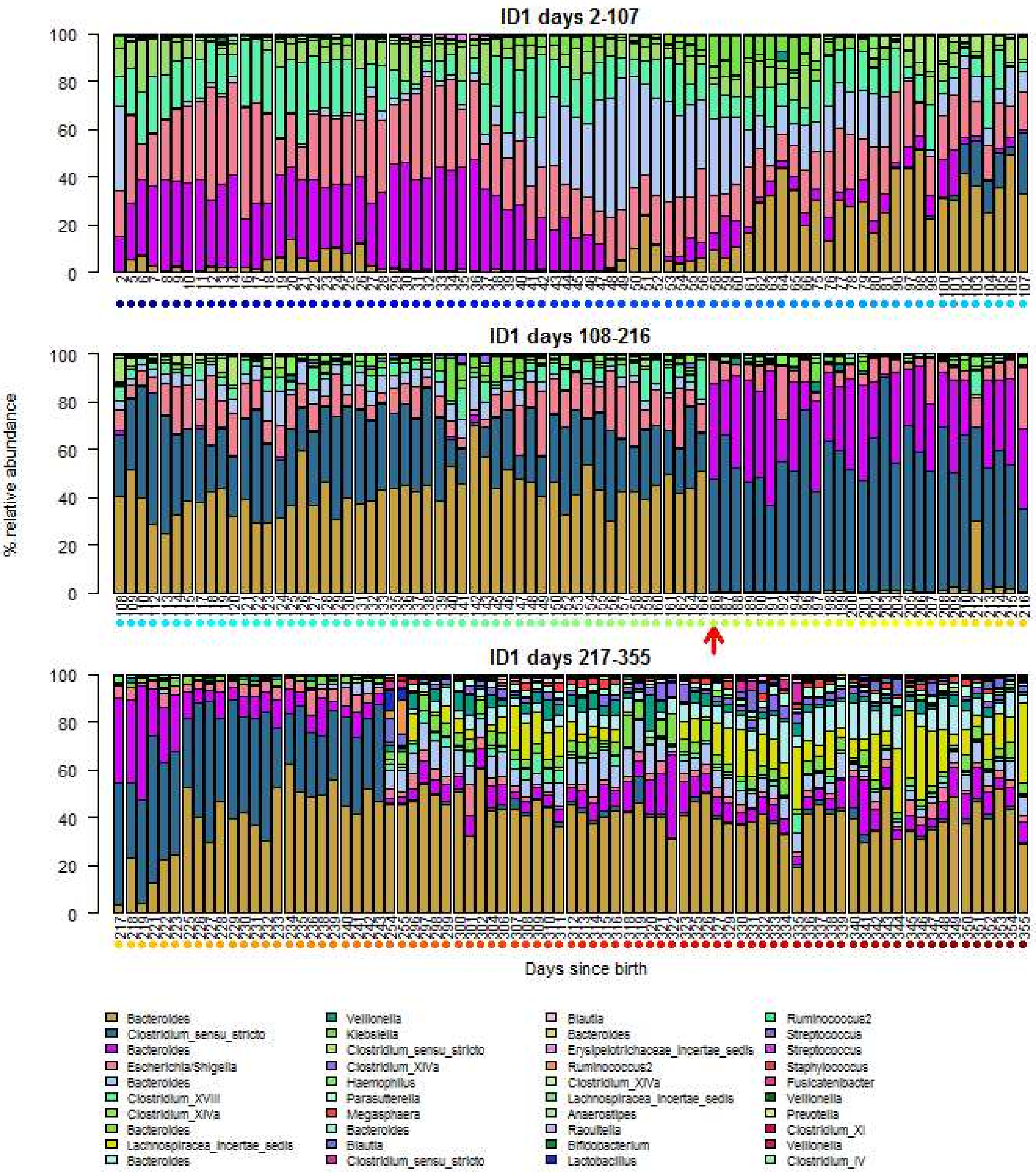

Supplementary Figure 4 | Relative abundances of the OTUs in ID1.
Relative abundances of the top 40 most abundant genera in ID1. Coloured dots under the day number correspond to the colour code displayed in Fig. 1. The red arrow indicates when the introduction of solid foods began. ID1 had periods of travel. These were during days 67-74, 167-185, 245-252, and 265-294. The colour key is consistent in Figures S4-S15 and in Figure 4 in the main text.

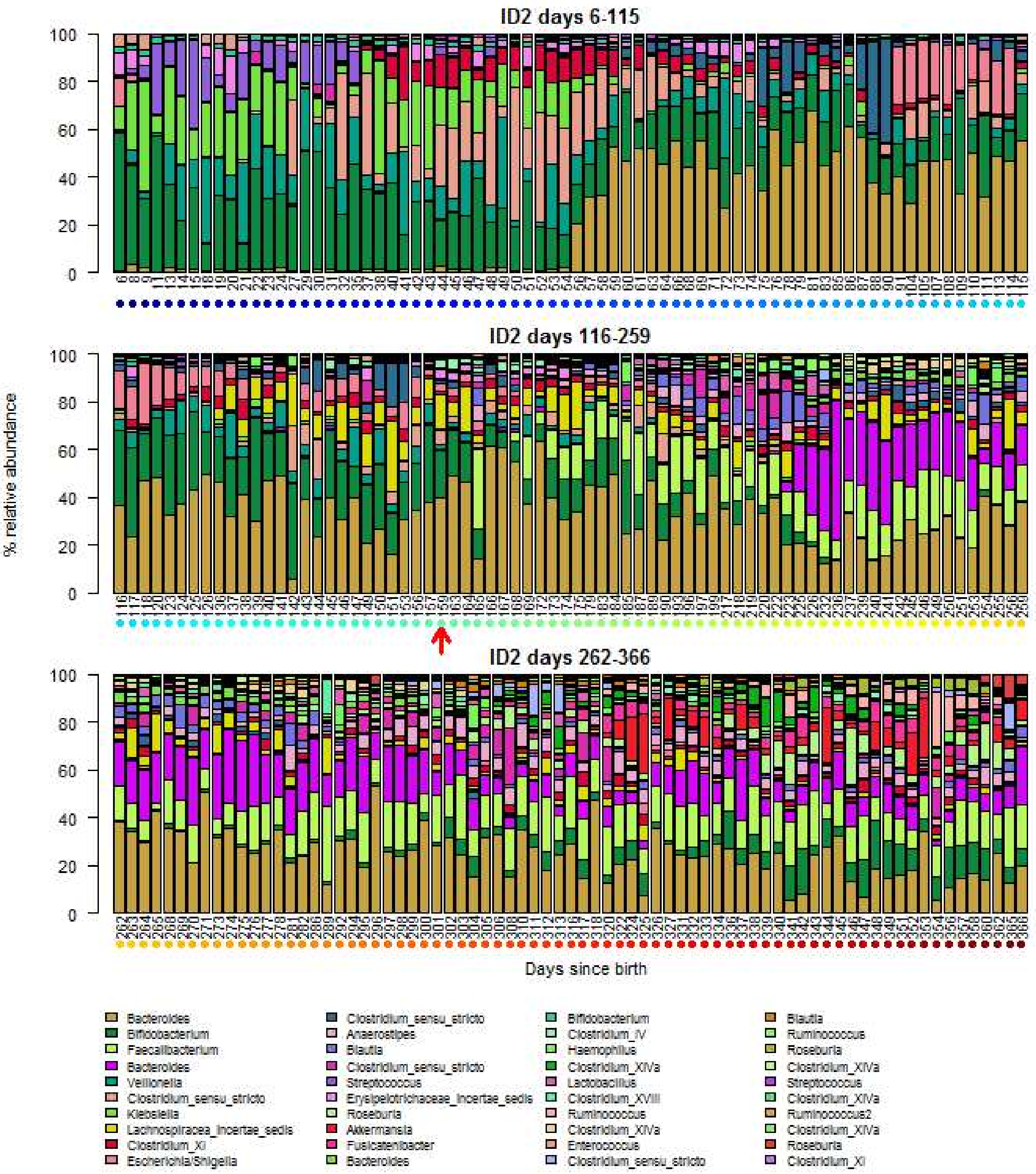

Supplementary Figure 5 | Relative abundances of the OTUs in ID2.
Relative abundances of the 40 most abundant genera in ID2. Coloured dots under the day number correspond to the colour code displayed in Fig. 1. The red arrow indicates when the introduction of solid foods began. ID2 had a period of travel during days 92-103. The colour key is consistent in Figures S4-S15 and in Figure 4 in the main text.

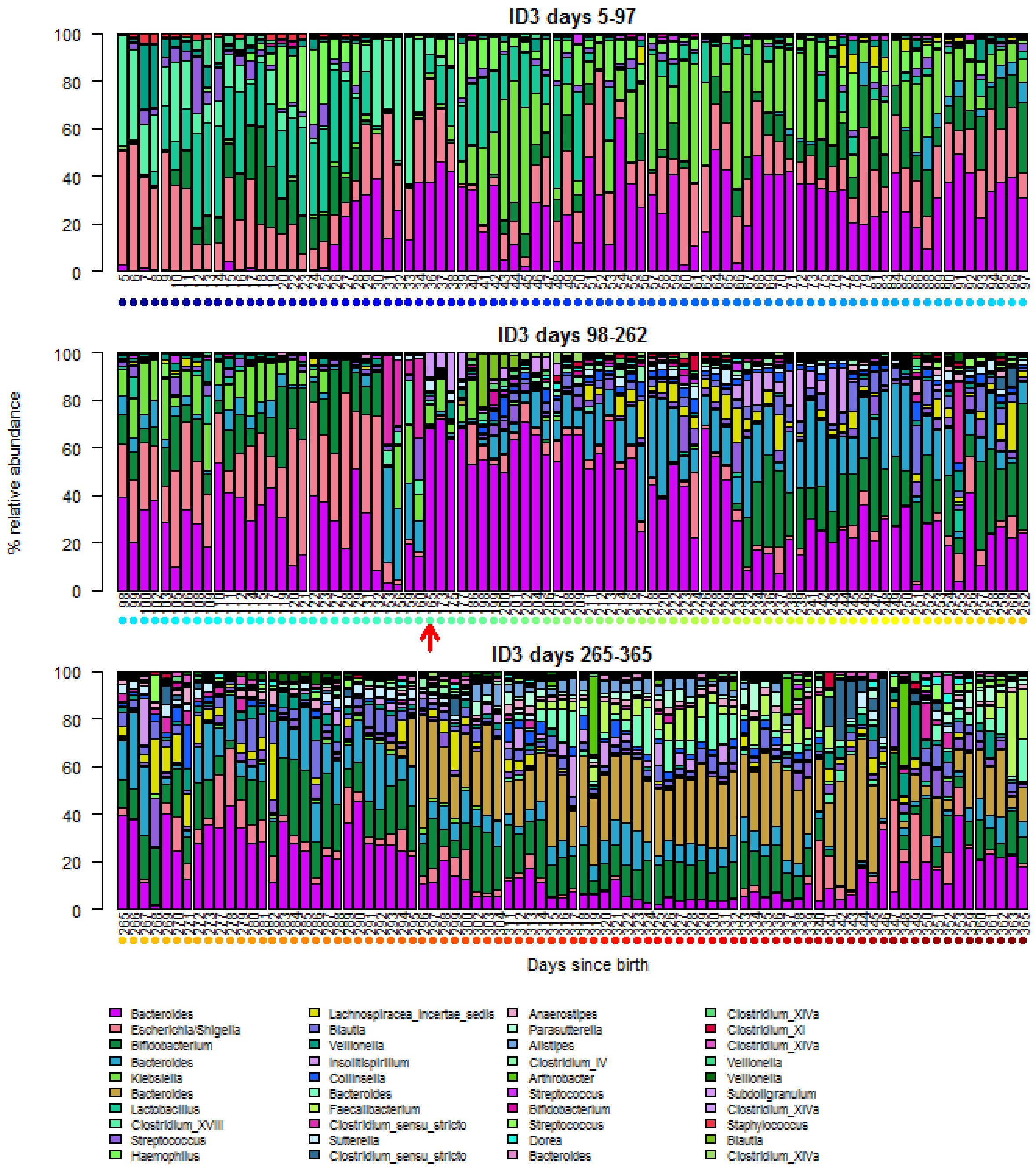

Supplementary Figure 6 | Relative abundances of the OTUs in ID3.
Relative abundances of the 40 most abundant genera in ID3. Coloured dots under the day number correspond to the colour code displayed in Fig. 1. The red arrow indicates when the introduction of solid foods began. ID3 had two periods of travel. These were between days 127-132 (sampled in 95% alcohol), 133-152 (no samples), 153-177 (sampled in 95% alcohol), 178-197 (sampled in 95% alcohol), 311-365 (sampled in 95% alcohol). The colour key is consistent in Figures S4-S15 and in Figure 4 in the main text.

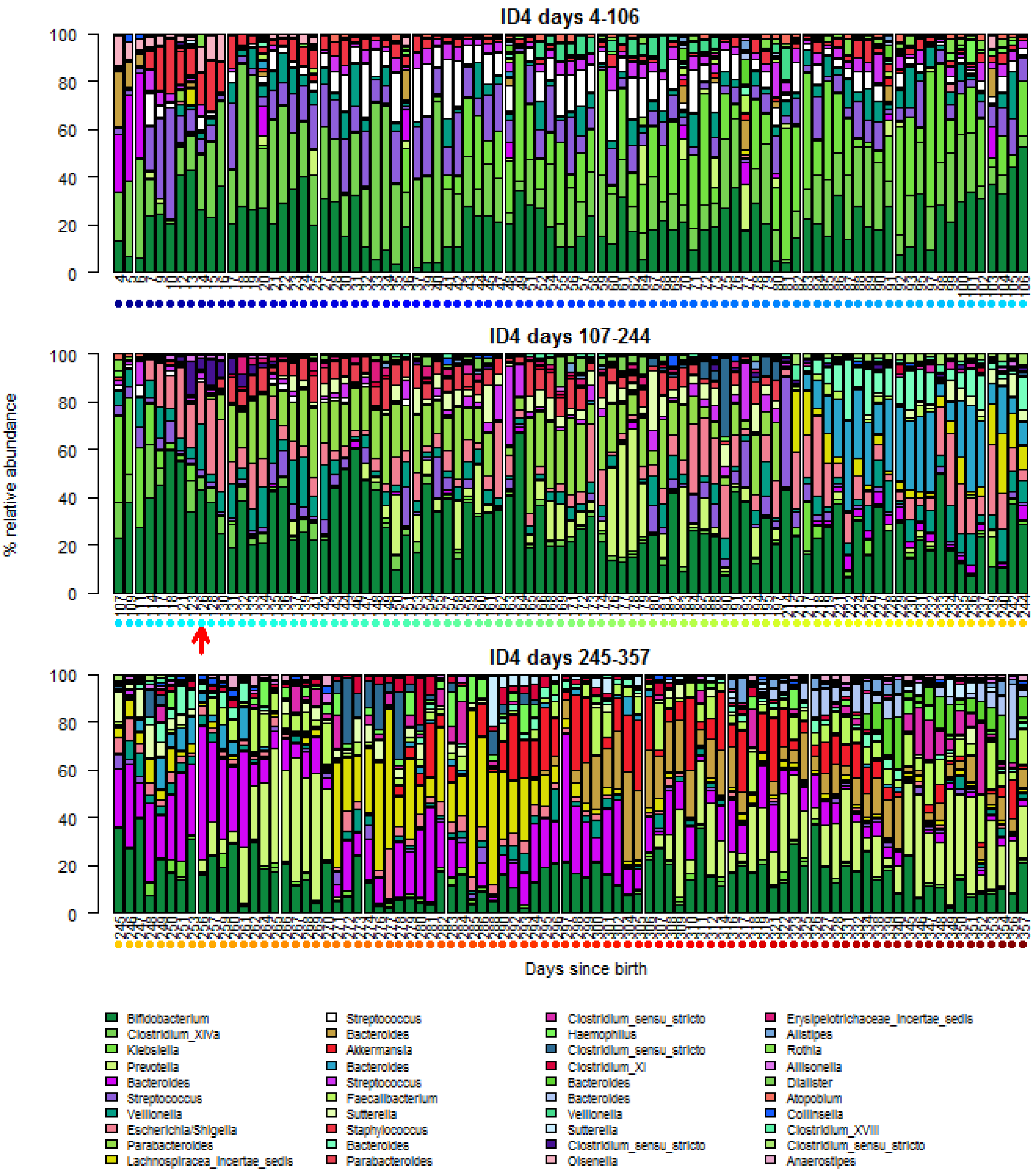

Supplementary Figure 7 | Relative abundances of the OTUs in ID4. Relative abundances of the 40 most abundant genera in ID4. Coloured dots under the day number correspond to the colour code displayed in Fig. 1. The red arrow indicates when the introduction of solid foods began. ID4 had a period of travel between days 198-213. The colour key is consistent in Figures S4-S15 and in Figure 4 in the main text.

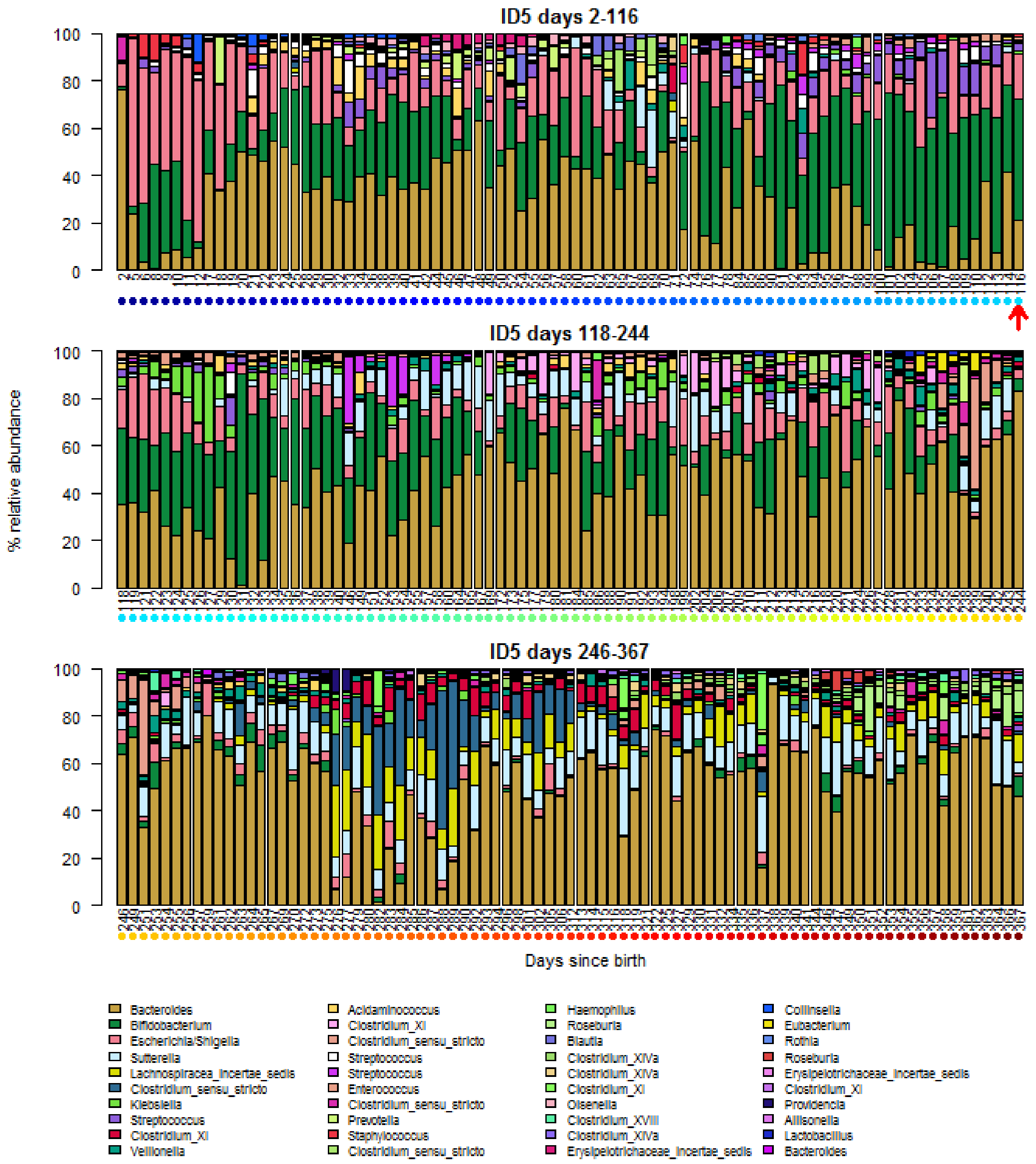

Supplementary Figure 8 | Relative abundances of the OTUs in ID5.
Relative abundances of the 40 most abundant genera in ID5. Coloured dots under the day number correspond to the colour code displayed in Fig. 1. The red arrow indicates when the introduction of solid foods began. ID5 had periods of travel between days 79-83, and 87-167 (sampled in 95% alcohol). The colour key is consistent in Figures S4-S15 and in Figure 4 in the main text.

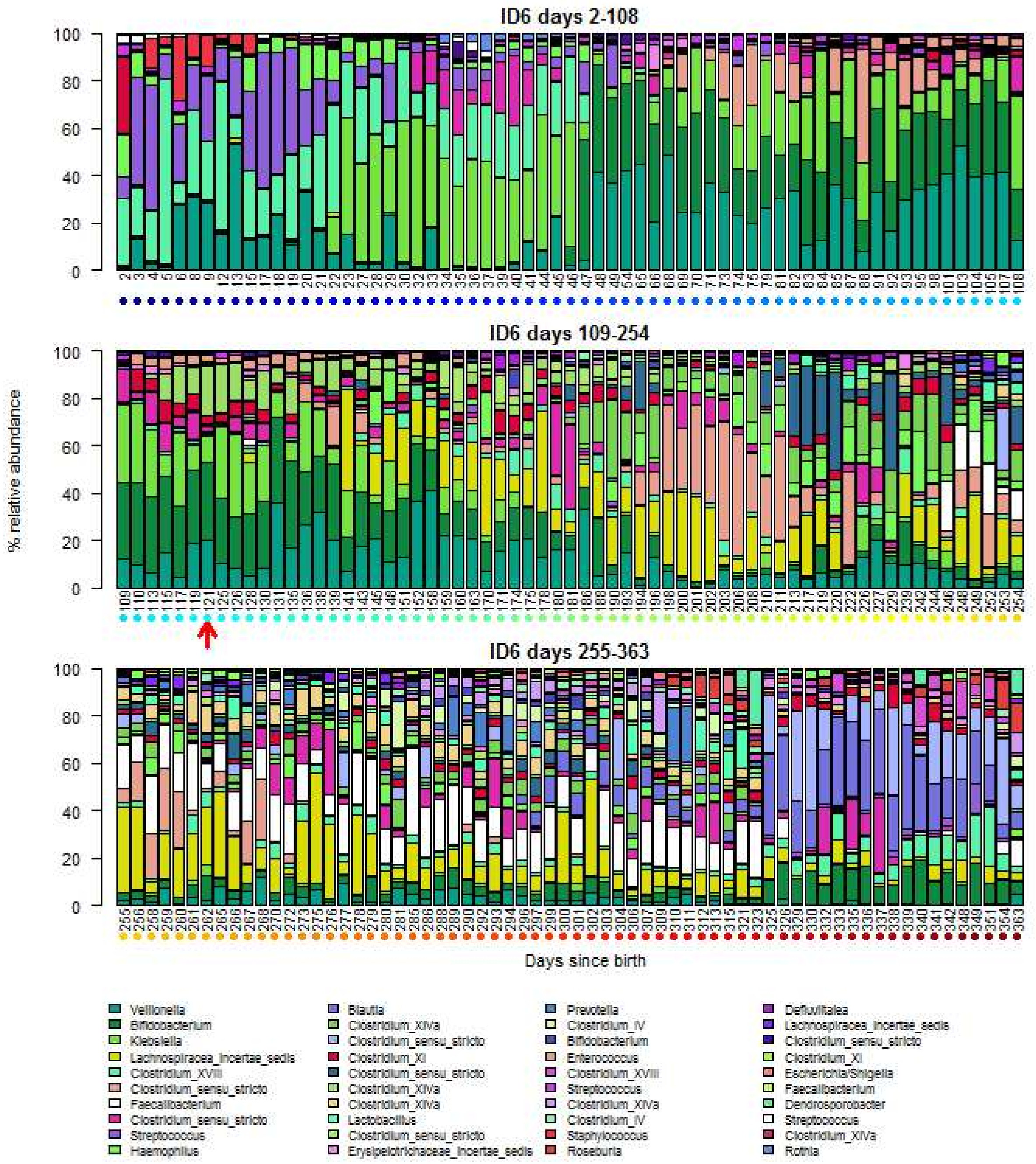

Supplementary Figure 9 | Relative abundances of the OTUs in ID6.

Relative abundances of the 40 most abundant genera in ID6. Coloured dots under the day number correspond to the colour code displayed in Fig. 1. The red arrow indicates when the introduction of solid foods began. The colour key is consistent in Figures S19-S27 and in Figure 4 in the main text. The colour key is consistent in Figures S4-S15 and in Figure 4 in the main text.

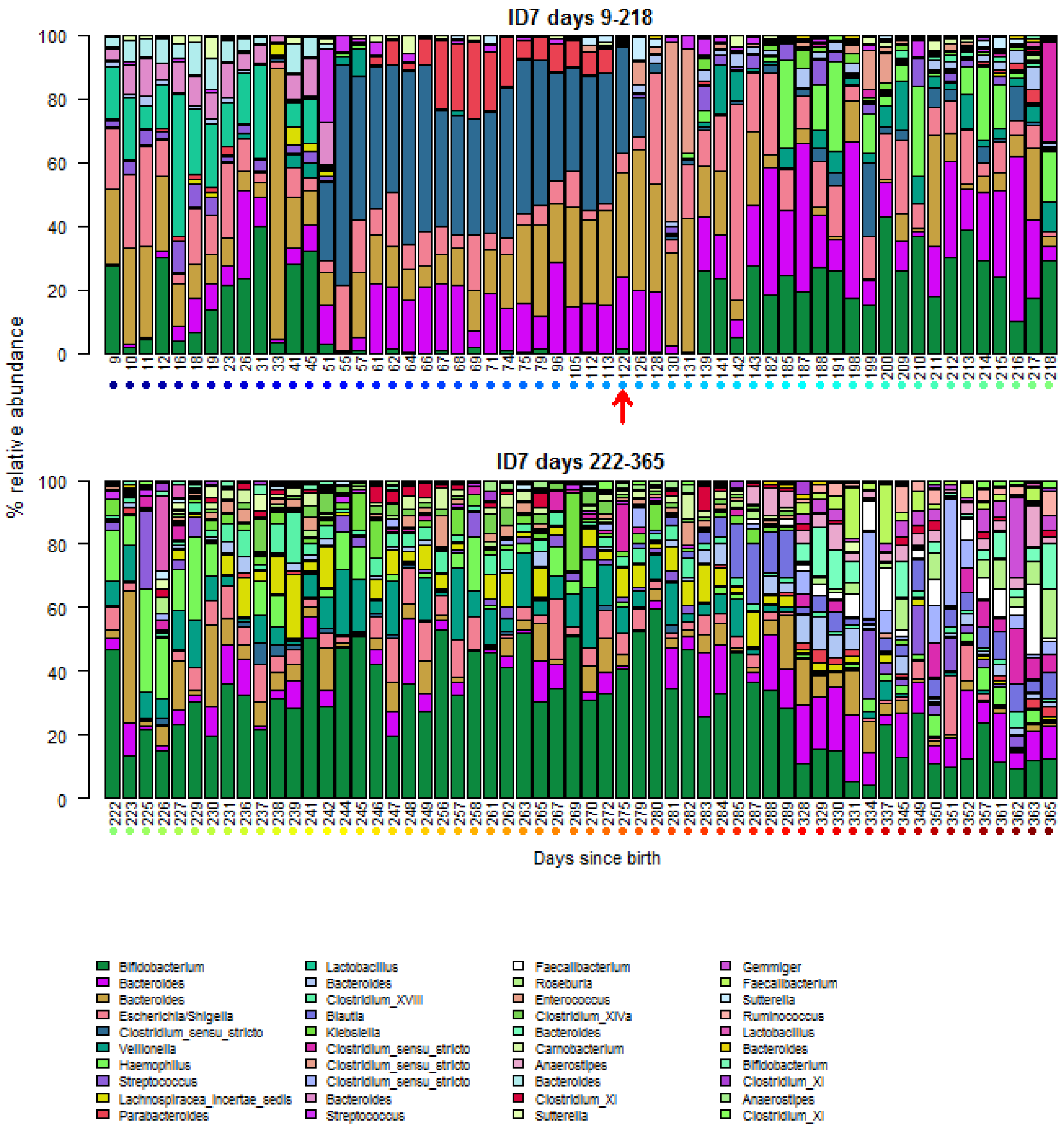

Supplementary Figure 10 | Relative abundances of the OTUs in ID7.
Relative abundances of the 40 most abundant genera in ID7. Coloured dots under the day number correspond to the colour code displayed in Fig. 1. The red arrow indicates when the introduction of solid foods began. ID7 had periods of travel during days 80-95, 144-181, and 290-327. The colour key is consistent in Figures S4-S15 and in Figure 4 in the main text.

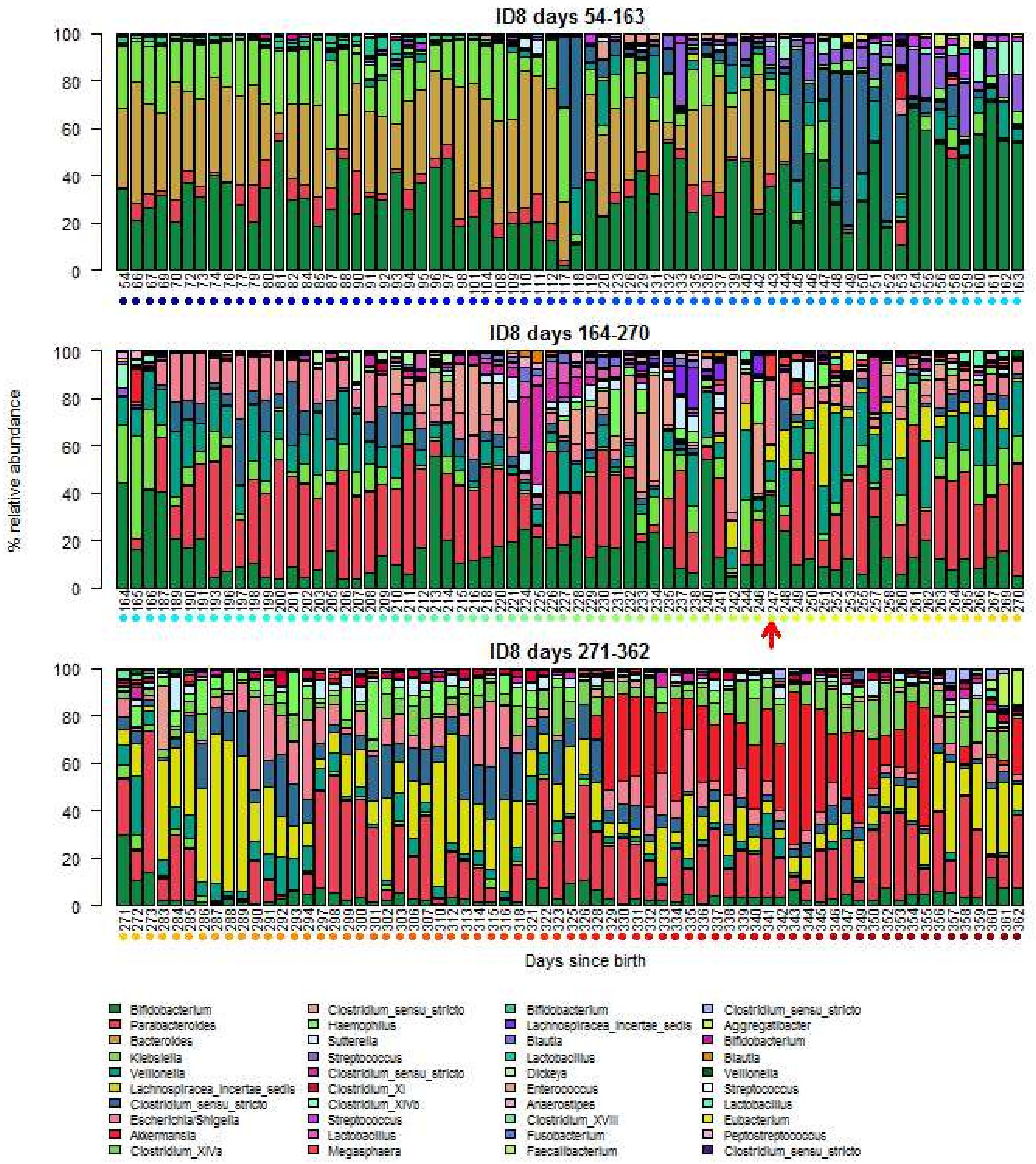

Supplementary Figure 11 | Relative abundances of the OTUs in ID8. Relative abundances of the 40 most abundant genera in ID8. Coloured dots under the day number correspond to the colour code displayed in Fig. 1. The red arrow indicates when the introduction of solid foods began. ID8 had periods of travel during days 167-183 and 274-282. The colour key is consistent in Figures S4-S15 and in Figure 4 in the main text.

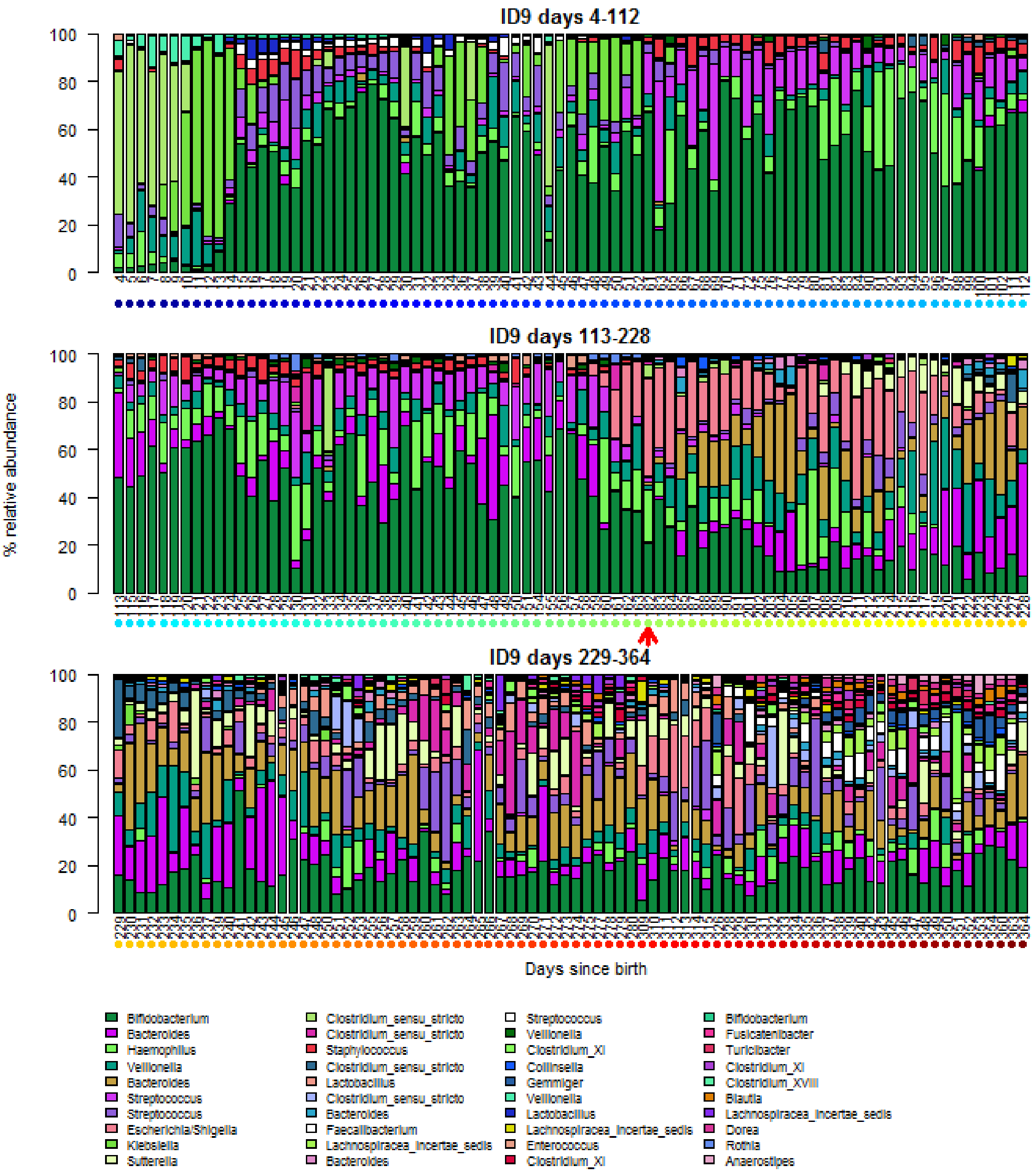

Supplementary Figure 12 | Relative abundances of the OTUs in ID9. Relative abundances of the 40 most abundant genera in ID9. Coloured dots under the day number correspond to the colour code displayed in Fig. 1. The red arrow indicates when the introduction of solid foods began. ID9 had periods of travel during days 53-60, 192-200, and 316-325. The colour key is consistent in Figures S4-S15 and in Figure 4 in the main text.

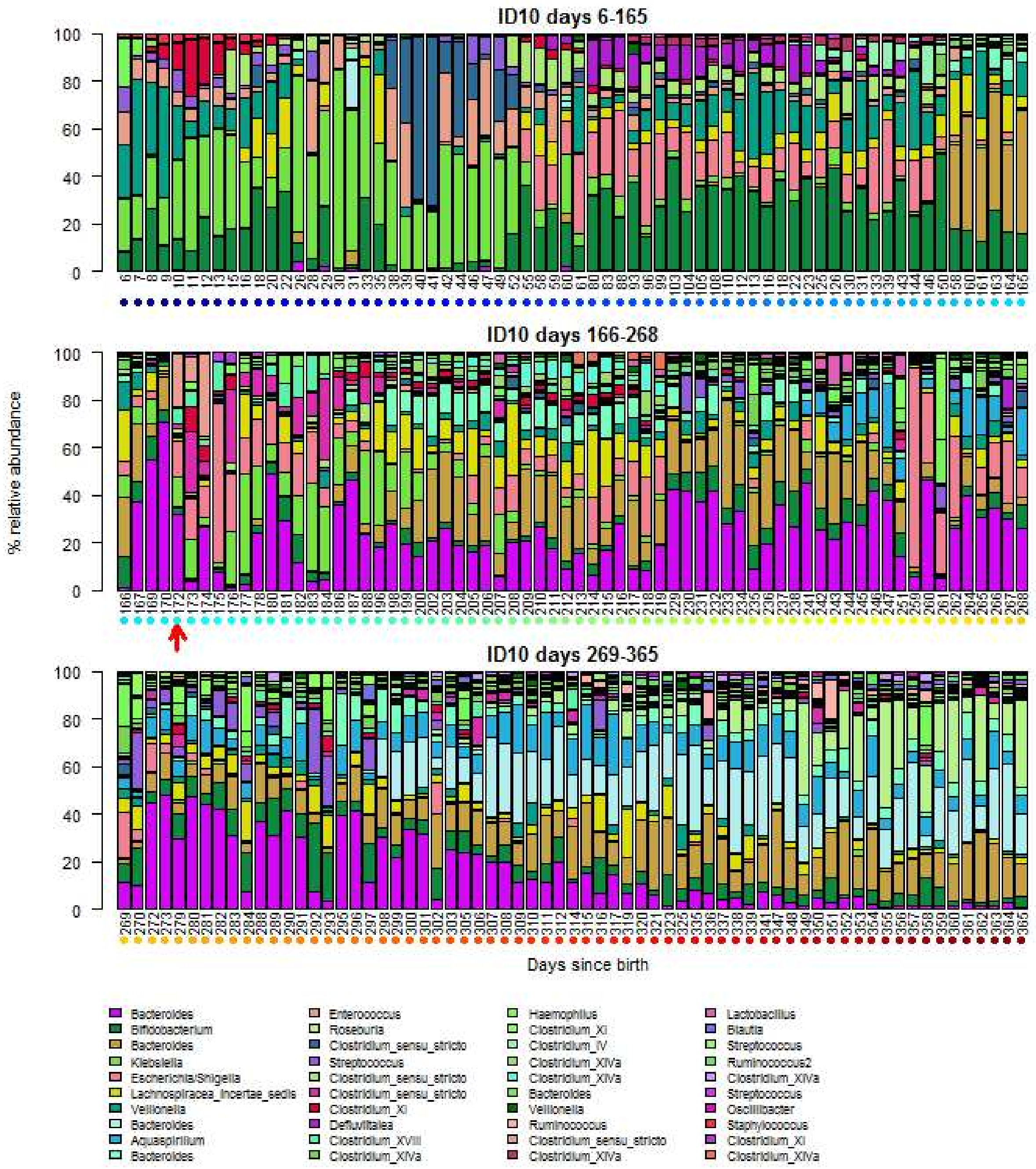

Supplementary Figure 13 | Relative abundances of the OTUs in ID10. Relative abundances of the 40 most abundant genera in ID10. Coloured dots under the day number correspond to the colour code displayed in Fig. 1. The red arrow indicates when the introduction of solid foods began. ID10 had periods of travel during days 80-170 (sampled in 95% alcohol), and 229-365 (sampled in 95% alcohol). The colour key is consistent in Figures S4-S15 and in Figure 4 in the main text.

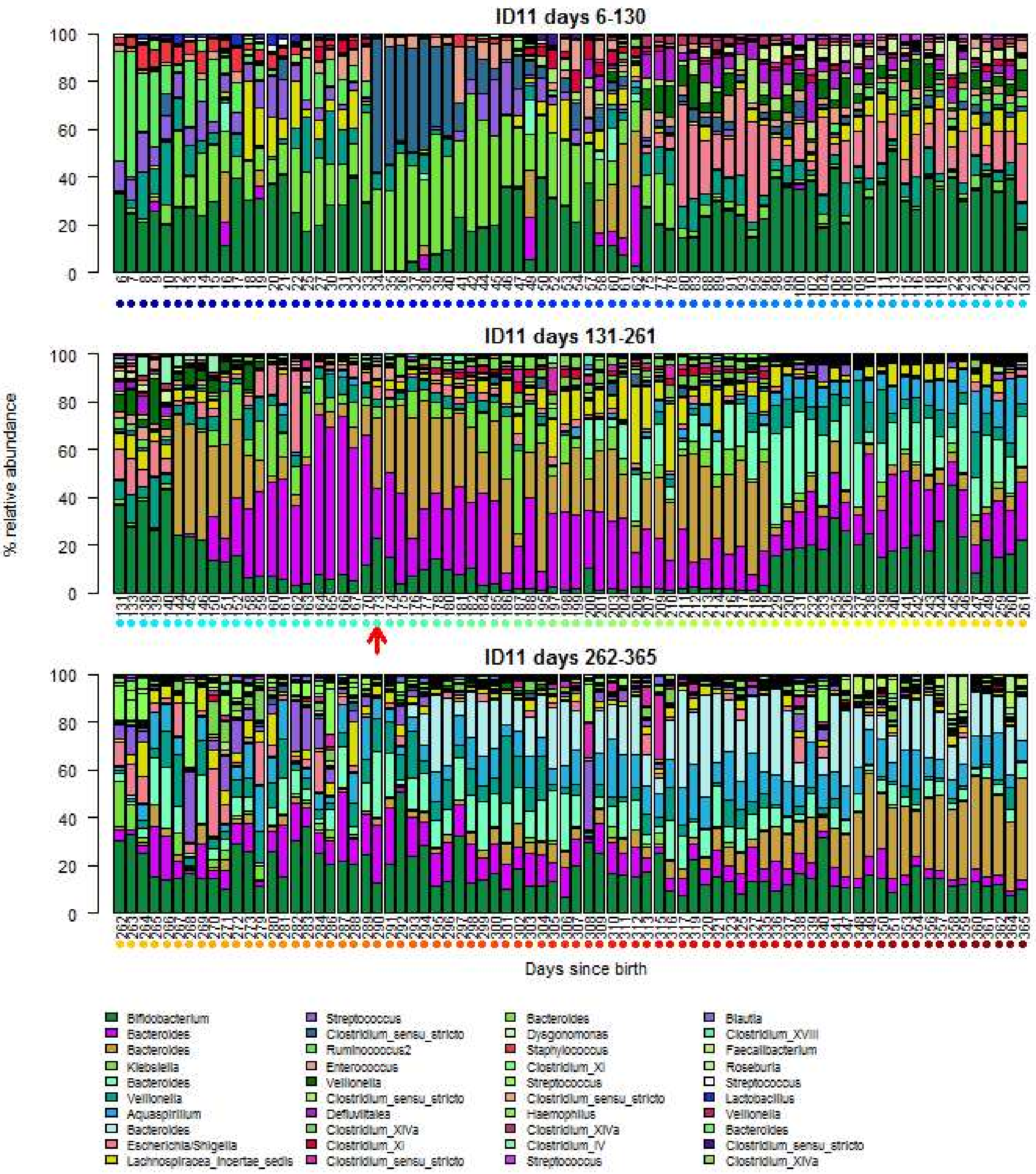

Supplementary Figure 14 | Relative abundances of the OTUs in ID11. Relative abundances of the 40 most abundant genera in ID11. Coloured dots under the day number correspond to the colour code displayed in Fig. 1. The red arrow indicates when the introduction of solid foods began. ID11 had periods of travel during days 80-170 (sampled in 95% alcohol), and 229-365 (sampled in 95% alcohol). The colour key is consistent in Figures S4-S15 and in Figure 4 in the main text.

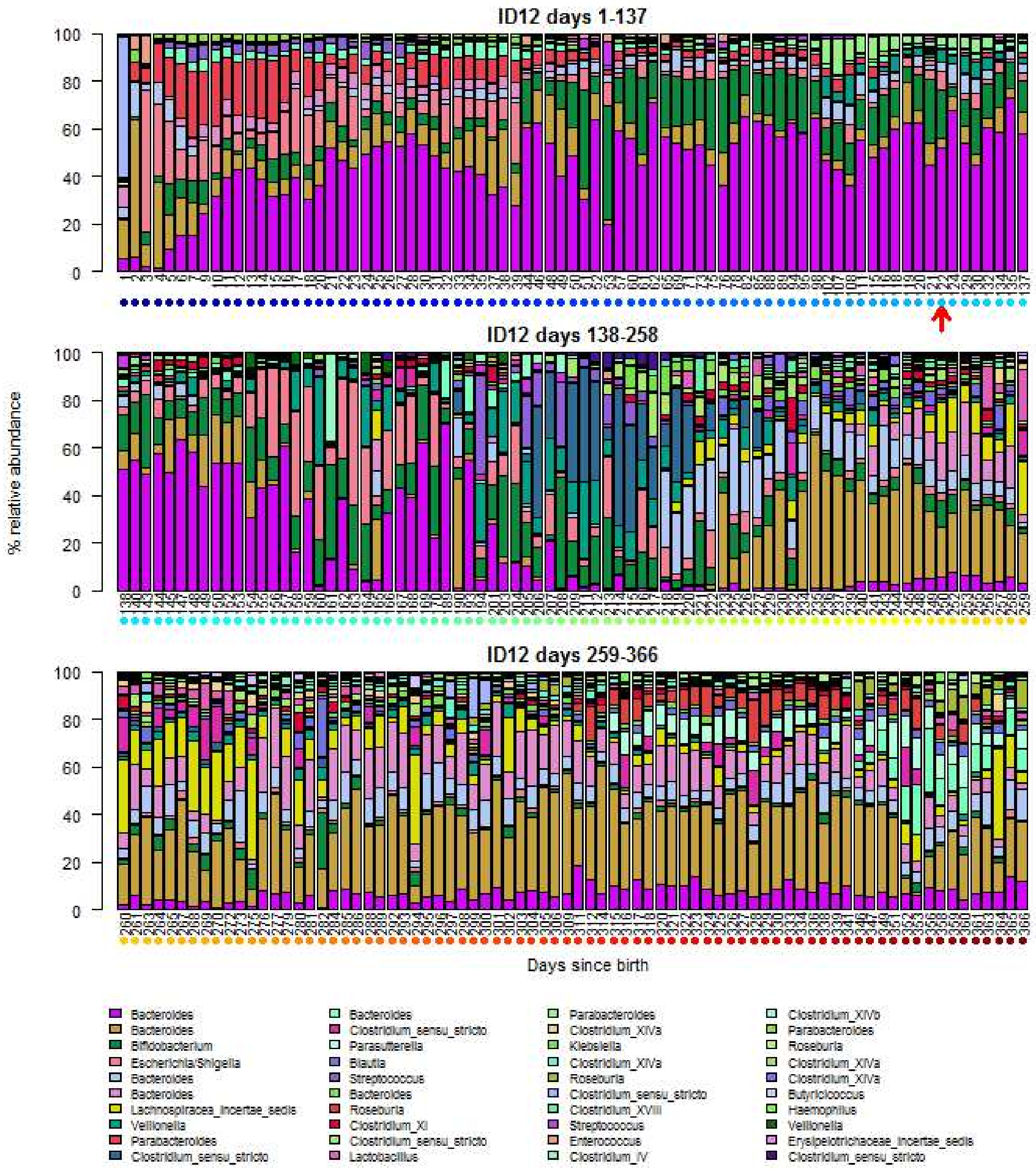

Supplementary Figure 15 | Relative abundances of the OTUs in ID12. Relative abundances of the 40 most abundant genera in ID12. Coloured dots under the day number correspond to the colour code displayed in Fig. 1. The red arrow indicates when the introduction of solid foods began. ID12 had a period of travel during days 171-188. The colour key is consistent in Figures S4-S15 and in Figure 4 in the main text.

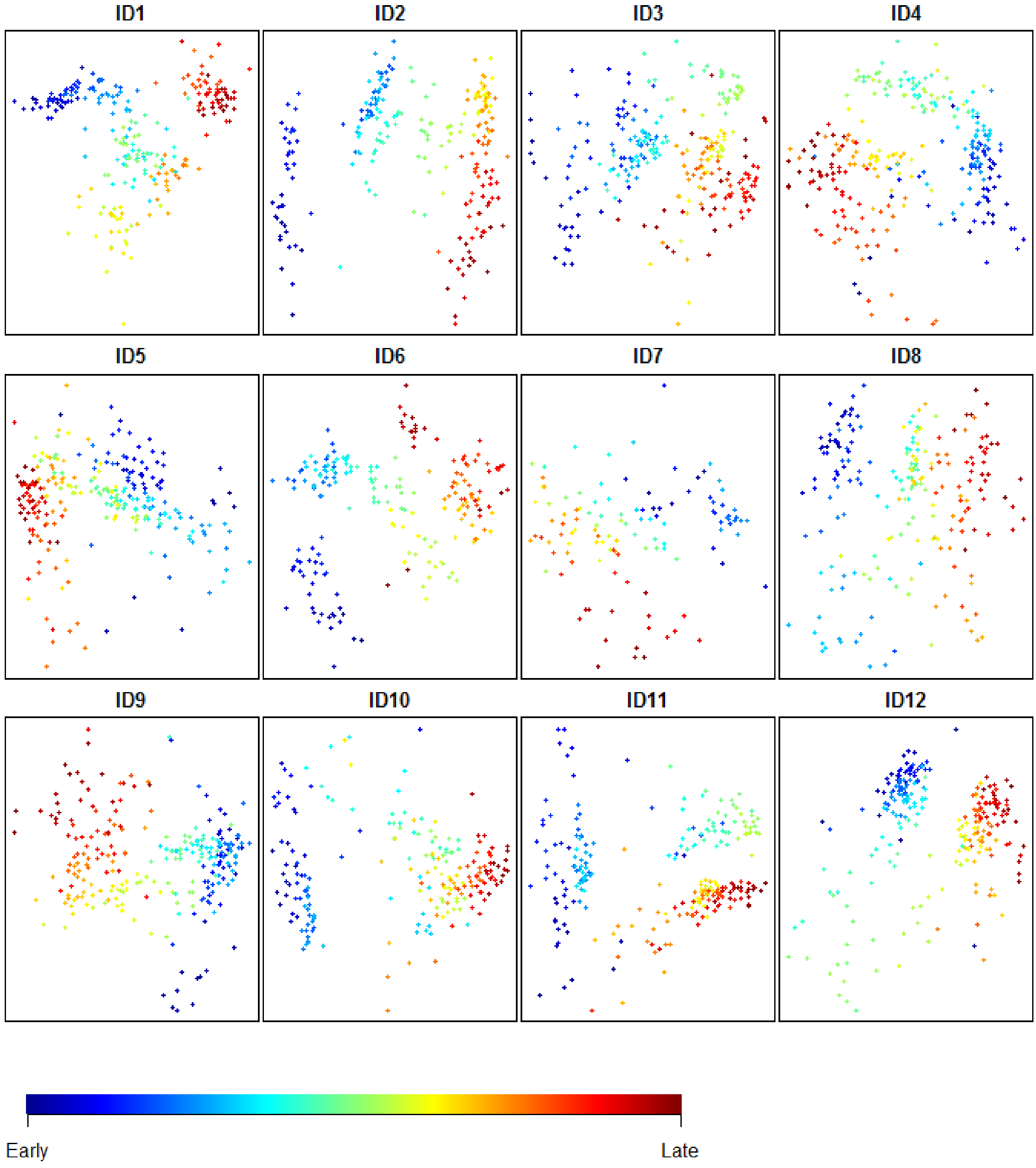

Supplementary Figure 16 | nMDS based on weighted UniFrac distances for all 12 infants. For each panel there is a highly significant relationship between the primary axis of variation and time since birth ($p<<0.001$ for all tests, mean $R^2=0.7$ (range 0.38-0.85), linear regression). Dots are coloured according to the bar beneath the panels. Early and late refer to the order in which the samples were collected (see Table 1 for days of first and last sampling).

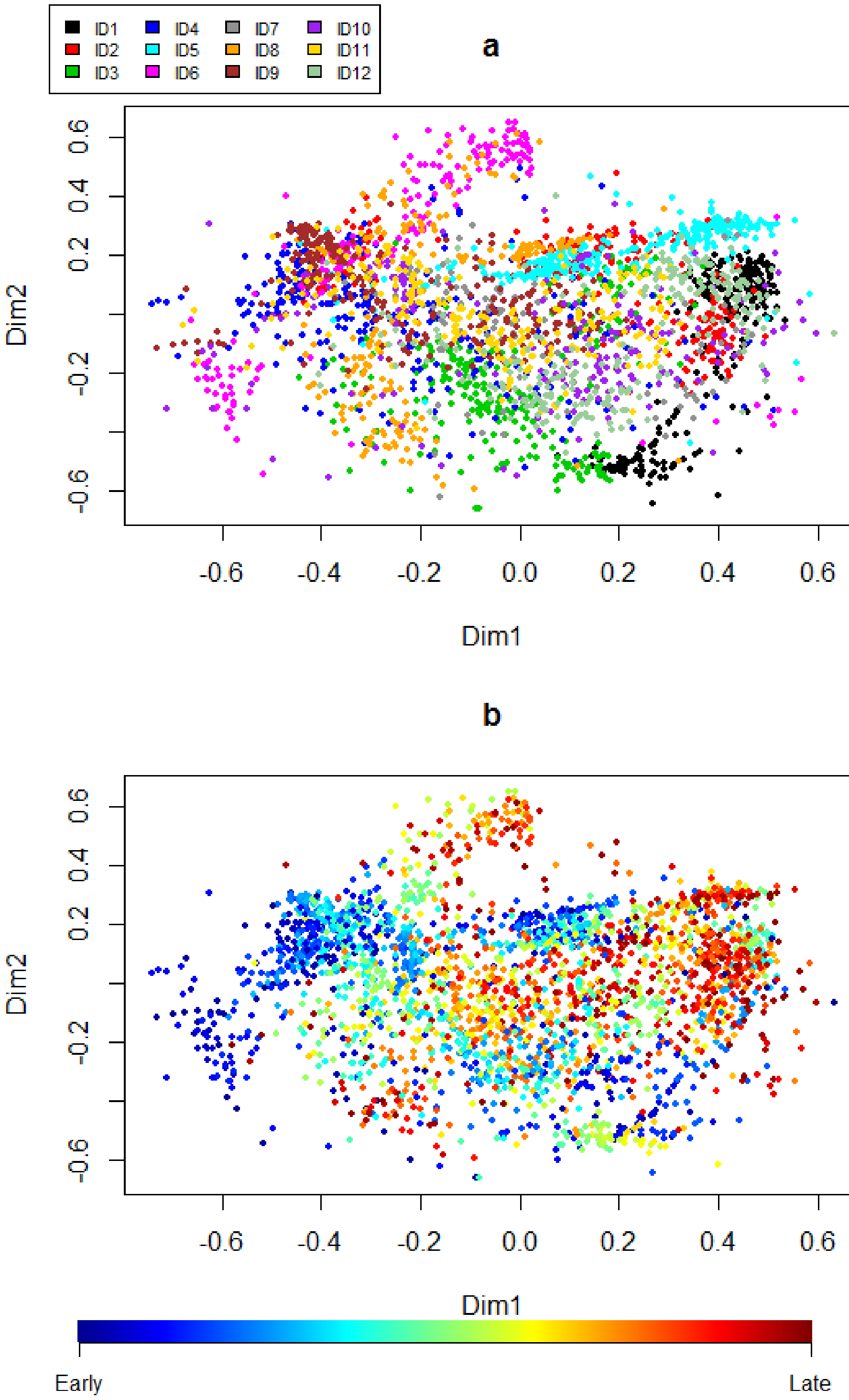

Supplementary Figure 17 | nMDS of all infants based on Bray-Curtis distances. Non-metric multidimensional scaling plot of all twelve infants using a single model. **a.** The infants showed significant clustering by individual ($p<0.001$, explained variance $R^2=0.29$; PERMANOVA). The colour code for each of the individual infants is indicated in the box above the panel. **b.** The infants showed a significant overall time trend when regressing the nMDS coordinates along the first dimension on time since birth ($p<0.001$, $R^2=0.17$, linear regression). For each infant, the colour code refers to the order in which the samples were collected (see Table 1 for days of first and last sampling). The models were based on Bray-Curtis distances.

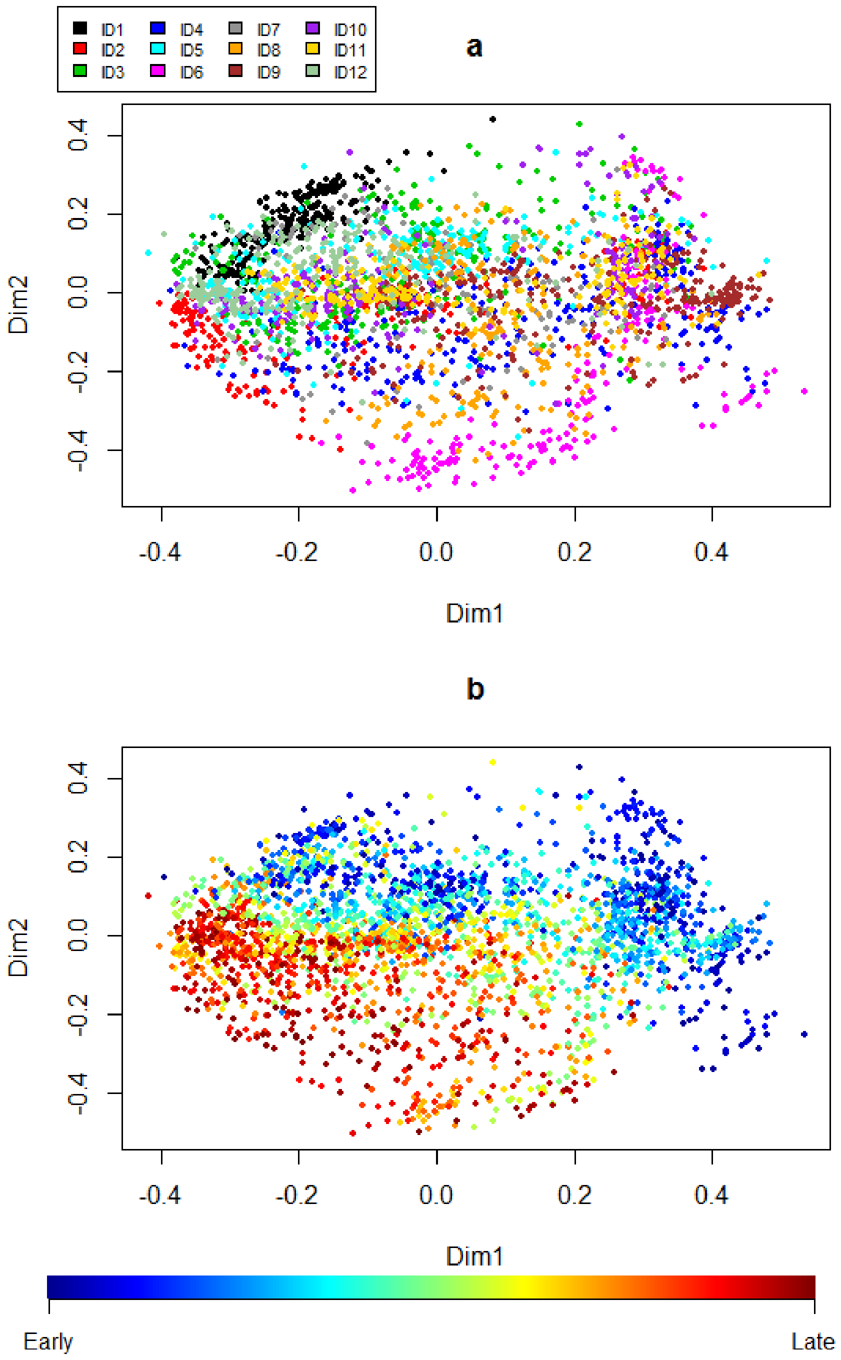

Supplementary Figure 18 | nMDS of all infants based on weighted UniFrac distances. Non-metric, multidimensional scaling plot of all twelve infants using a single model. **a.** The infants showed significant clustering by individual ($p<0.001$, explained variance $R^2=0.27$; PERMANOVA). The colour code for each of the individual infants is indicated in the box above the panel. **b.** The infants showed a significant overall time trend when regressing the nMDS coordinates along the first dimension on time since birth ($p<0.001$, $R^2=0.26$, linear regression). The bar beneath the panel indicates the time since birth when a sample was collected. The time trend was even stronger when looking at the second dimension ($p<0.001$, $R^2=0.3$). For each infant, the colour code refers to the order in which the samples were collected (see Table 1 for days of first and last sampling).

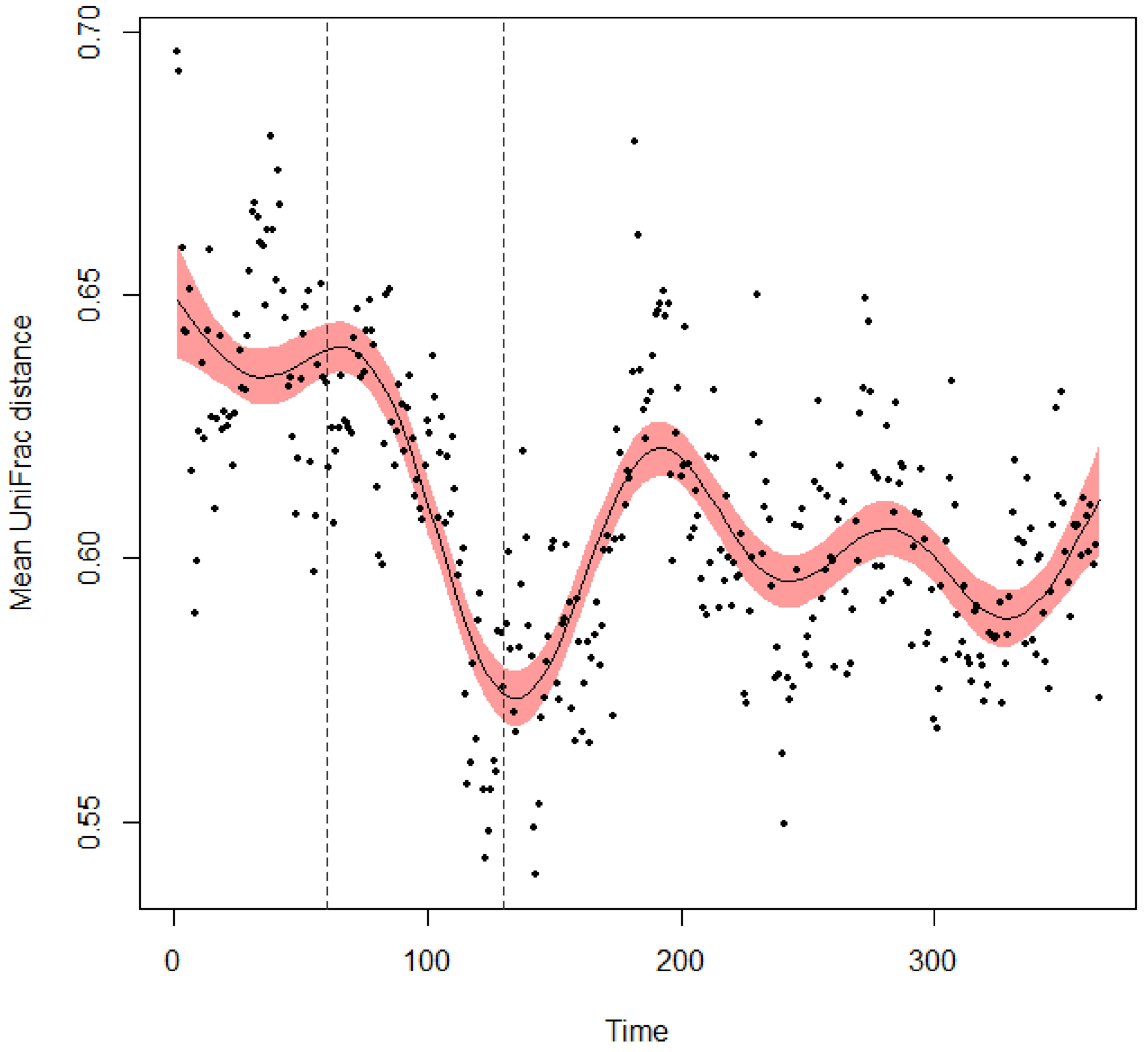

Supplementary Figure 19 | Mean pairwise contemporaneous weighted UniFrac distances between 11 infants*. All data series were interpolated to equal length (365 pseudo-days) for this analysis. Dots show the observed values while the black line shows a fitted generalized additive model ($p<<0.001$, $R^2=0.53$). The shaded band represents 95% confidence limits. Dotted vertical lines indicate the window of convergence (days 60-130). * ID8 was excluded from this analysis since sampling did not commence until day 54.

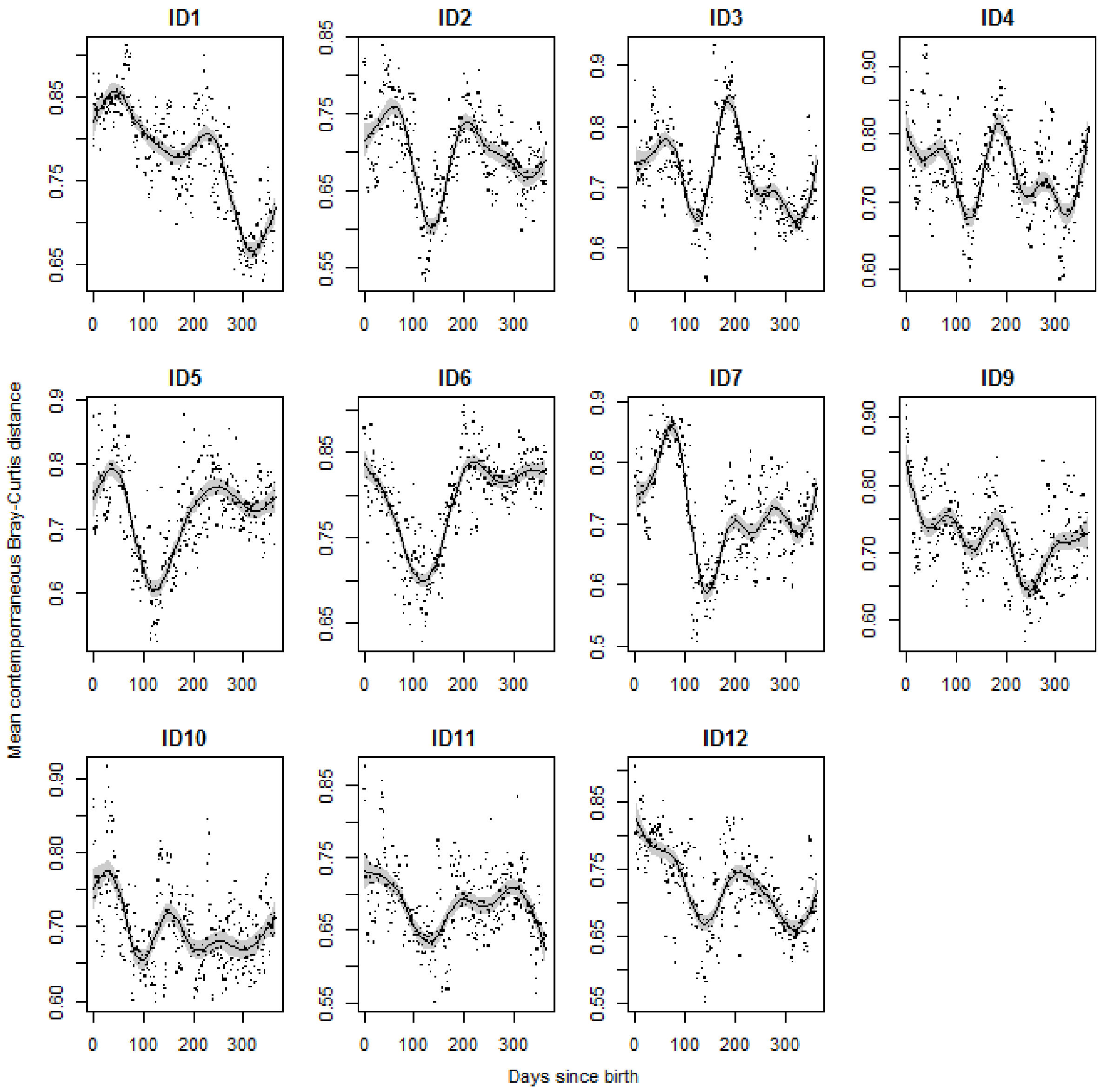

Supplementary Figure 20 | Mean contemporaneous Bray-Curtis distances. In each panel the dots represent the mean distance between the individual indicated above and 10 other individuals over time*, based on data series interpolated to 365 points. The fitted lines are generalized additive models using 9 degrees of freedom for estimating the smooth terms. Shaded areas represent 95% confidence limits. *ID8 was excluded from this analysis since sampling did not commence until day 54.

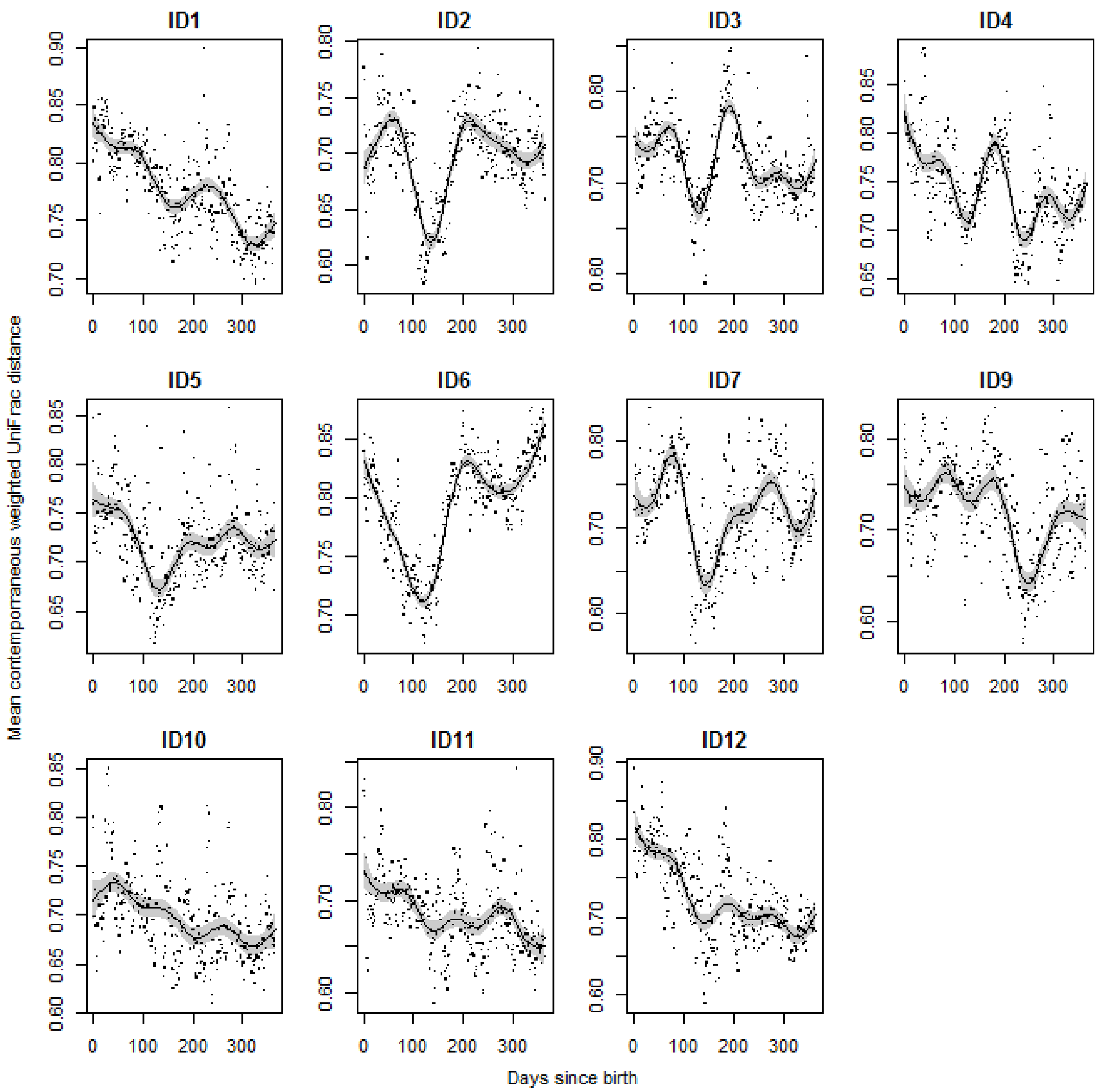

Supplementary Figure 21 | Mean contemporaneous weighted UniFrac distances. In each panel the dots represent the mean distance between the individual indicated above and 10 other individuals over time*, based on data series interpolated to 365 points. The fitted lines are generalized additive models using 9 degrees of freedom for estimating the smooth terms. Shaded areas represent 95% confidence limits. *ID8 was excluded from this analysis since sampling did not commence until day 54.

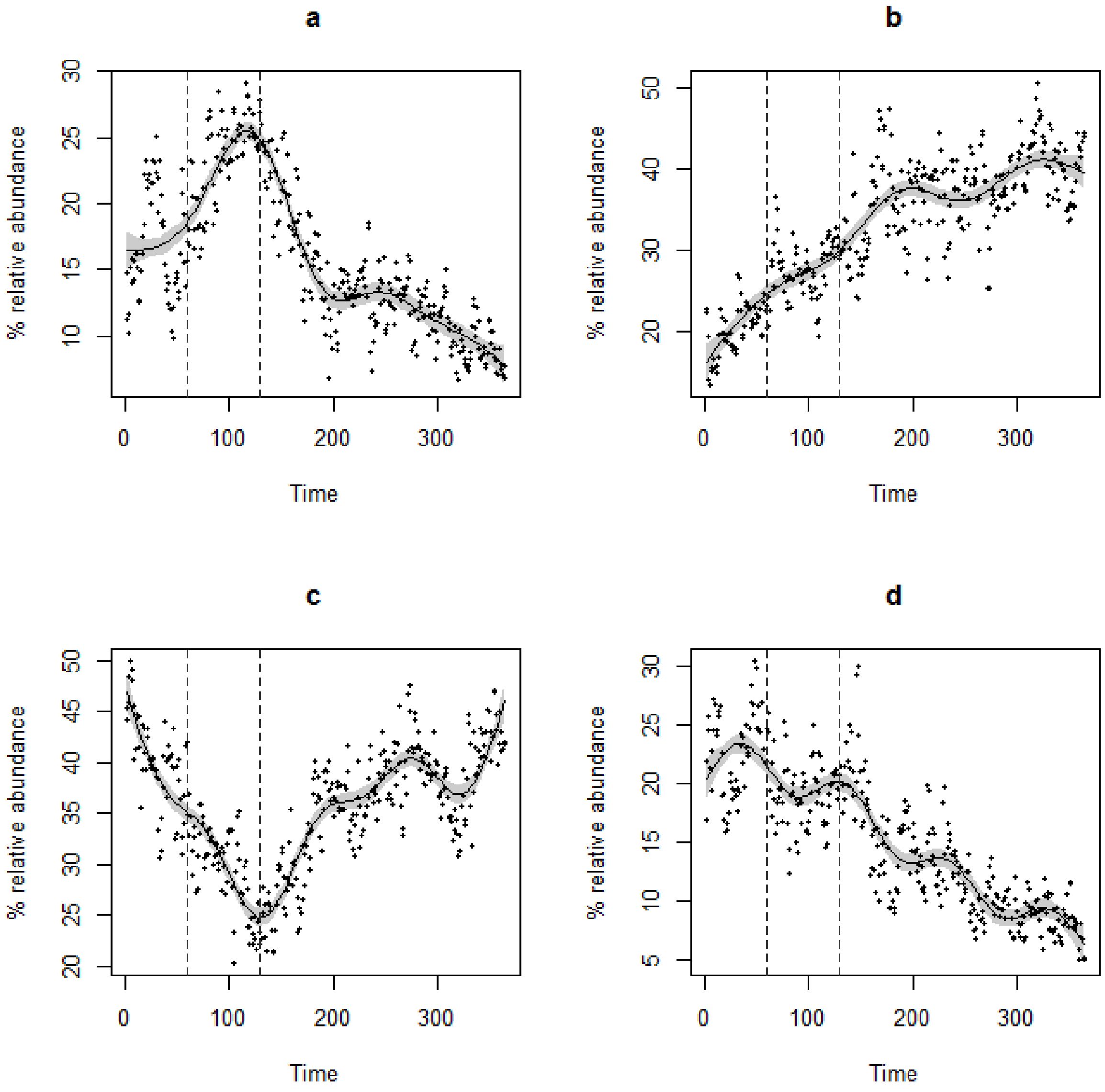

Supplementary Figure 22 | Mean phylum level relative abundances. Mean relative abundances of the interpolated series (365 data points) of the four main phyla in 11 of the infants*. **(a)** Actinobacteria, **(b)** Bacteroidetes, **(c)** Firmicutes, **(d)** Proteobacteria. The fitted lines are generalized additive models using 9 degrees of freedom for estimating the smooth terms. Shaded areas represent 95% confidence limits. Dotted vertical lines indicate the period of convergence (days 60 to 130). *ID8 was excluded from this analysis since sampling did not commence until day 54.

‘

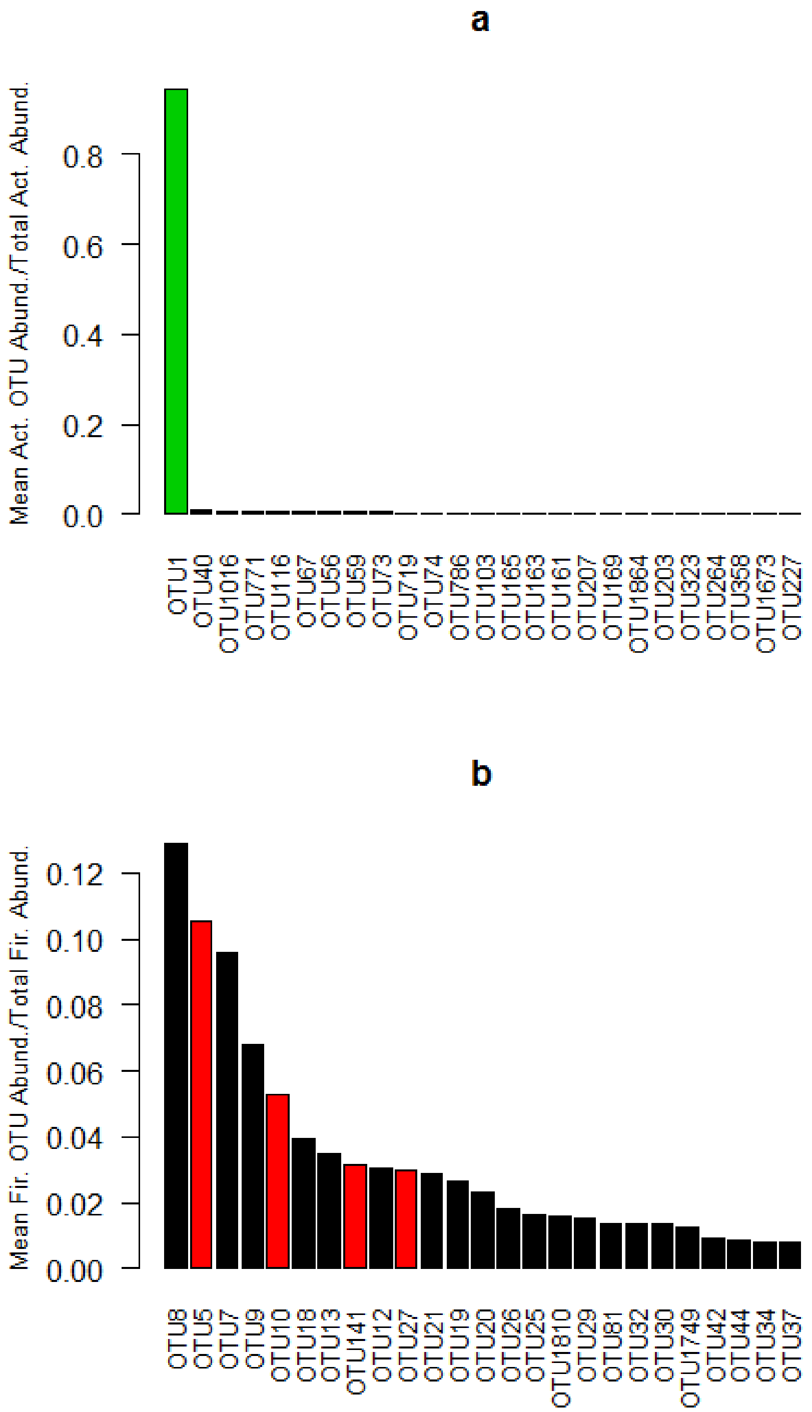

Supplementary Figure 23 | Within-phylum OTU proportions. a,b, Ratios of the relative abundances of the 25 most abundant OTUs classified as Actinobacteria (a) and Firmicutes (b) to the total relative abundances of Actinobacteria and Firmicutes, respectively. Coloured bars correspond to OTUs with a significant negative (green) or positive (red) correlation with mean contemporaneous Bray-Curtis distances during the convergence period (days 60-130) (Table S2). Act. = Actionbacteria, Fir. = Firmicutes. Abund. = Abundance.

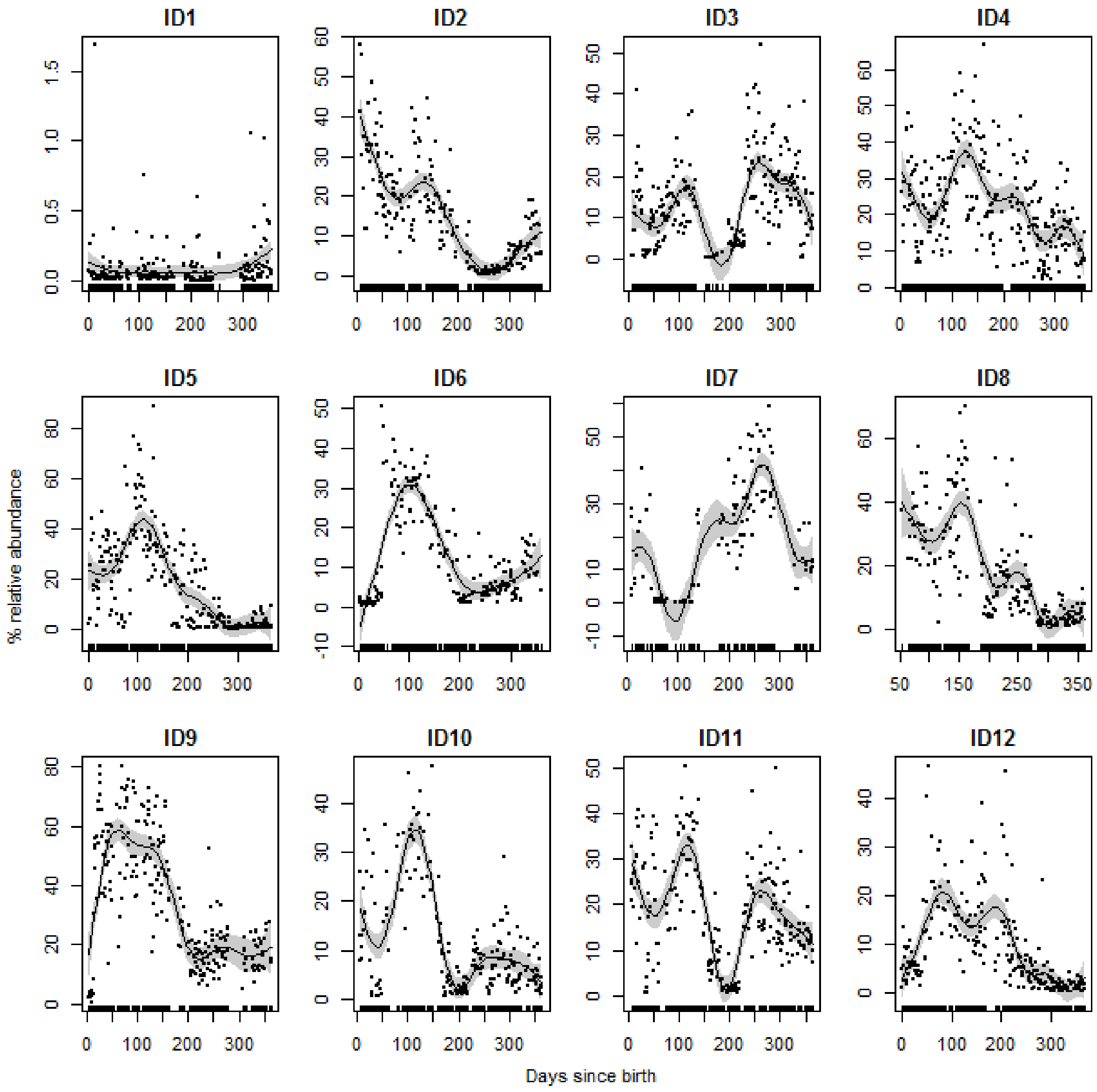

Supplementary Figure 24 | Relative abundance of Actinobacteria.
Relative abundances of Actinobacteria through the first year of life for the twelve infants. The fitted lines are generalized additive models using 9 degrees of freedom for estimating the smooth terms. Shaded areas represent 95% confidence limits. The rugs along the x-axis indicate days for which we had samples.

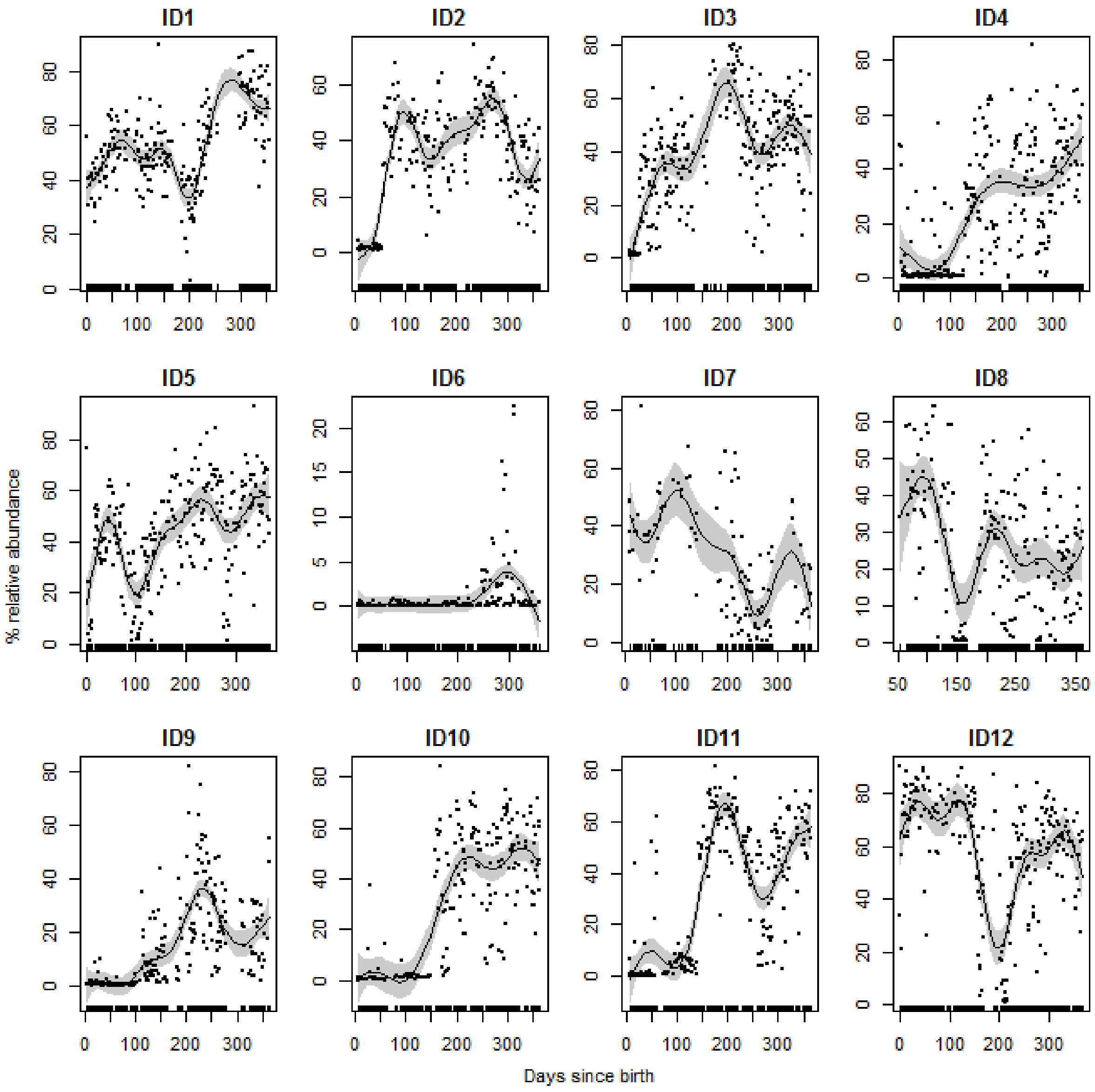

Supplementary Figure 25 | Relative abundance of Bacteroidetes.

Relative abundances of Bacteroidetes through the first year of life for the twelve infants. The fitted lines are generalized additive models using 9 degrees of freedom for estimating the smooth terms. Shaded areas represent 95% confidence limits. The rugs along the x-axis indicate days for which we had samples.

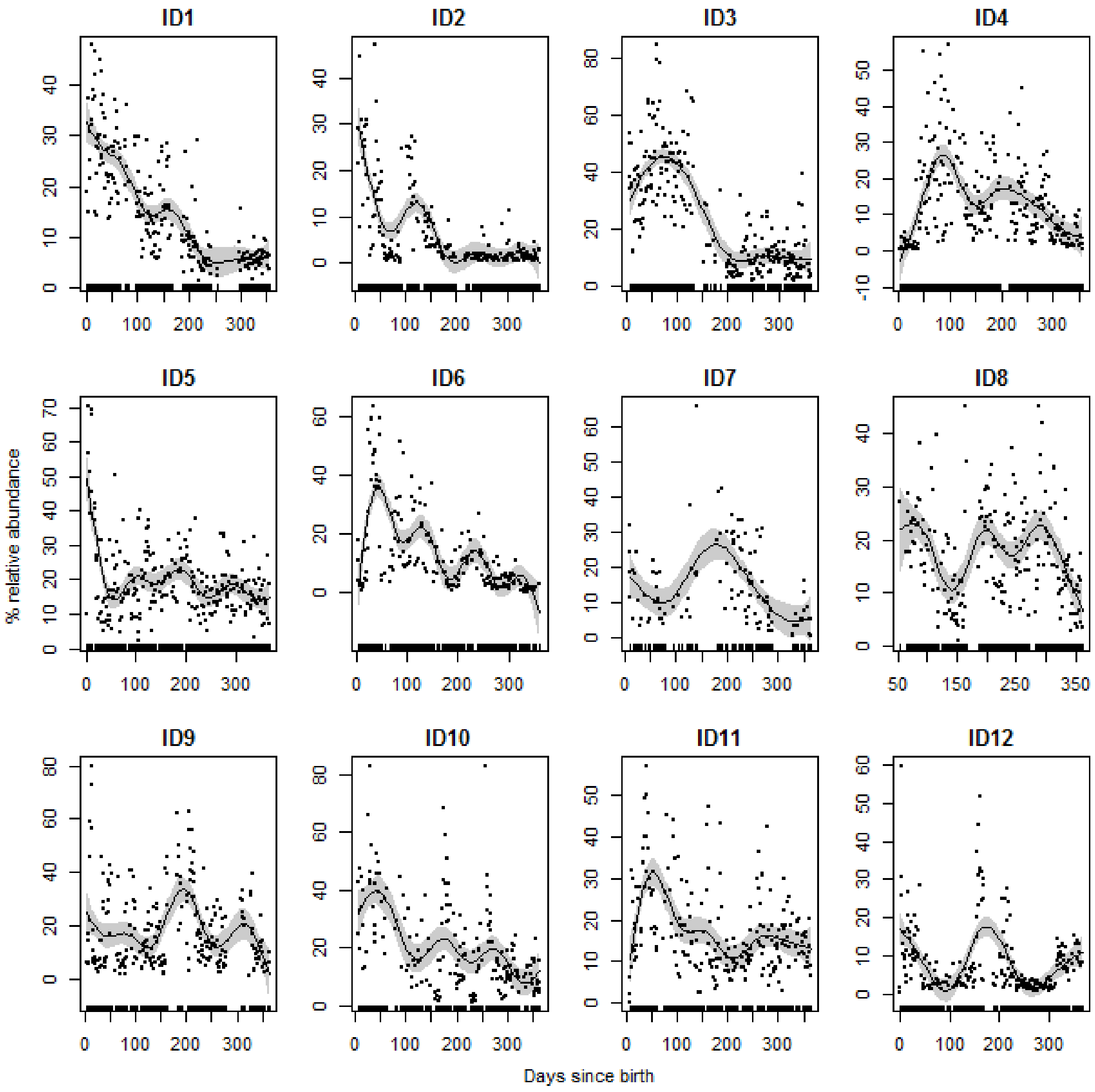

Supplementary Figure 26 | Relative abundance of Proteobacteria.
Relative abundances of Proteobacteria through the first year of life for the twelve infants. The fitted lines are generalized additive models using 9 degrees of freedom for estimating the smooth terms. Shaded areas represent 95% confidence limits. The rugs along the x-axis indicate days for which we had samples.

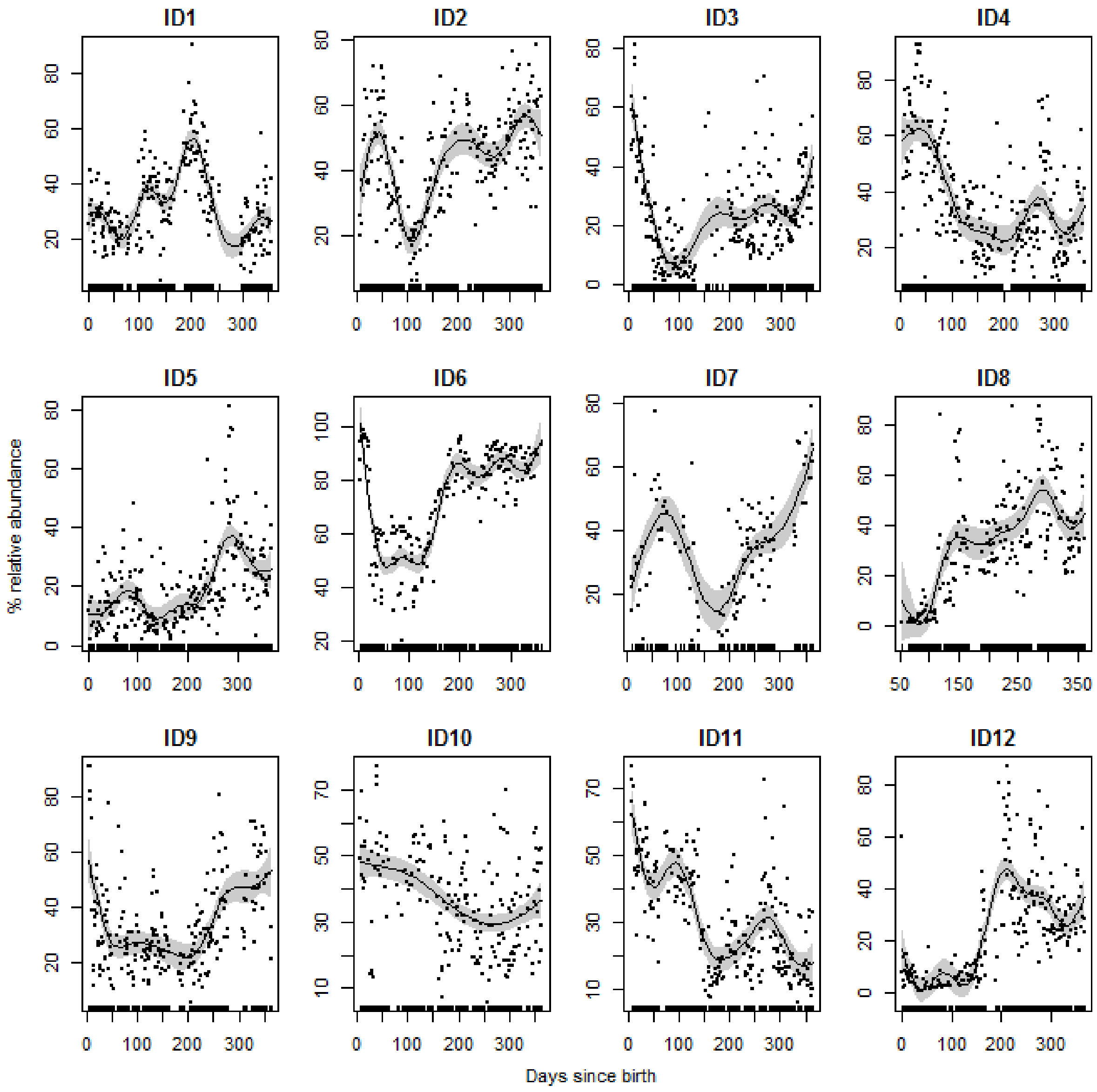

Supplementary Figure 27 | Relative abundance of Firmicutes.
Relative abundances of Firmicutes through the first year of life for the twelve infants. The fitted lines are generalized additive models using 9 degrees of freedom for estimating the smooth terms. Shaded areas represent 95% confidence limits. The rugs along the x-axis indicate days for which we had samples.

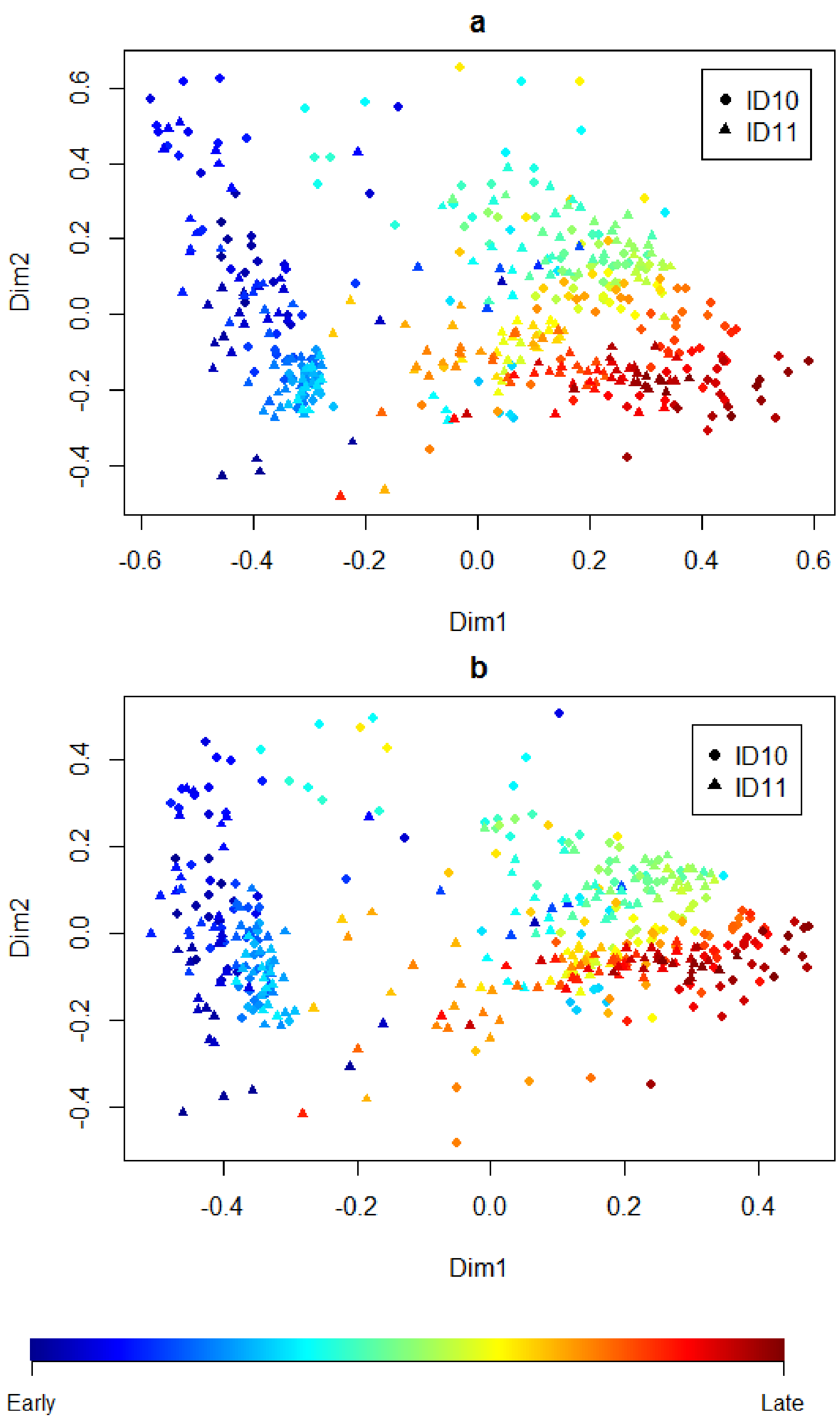

Supplementary Figure 28 | nMDS of samples from both twins.
Non-metric, multidimensional scaling of all samples from ID10 (n=203) and ID11 (n=234). **a.** Model based on Bray-Curtis distances. The primary axis of variation was highly correlated with time since birth (p<<0.001, mean R^2=0.72, linear regression). **b.** Model based on unweighted UniFrac distances. The primary axis of variation was highly correlated with time since birth (p<<0.001, mean R^2=0.68, linear regression). Symbols are coloured according to the bar beneath the panels. Early and late refer to the order in which the samples were collected (see Table 1 for days of first and last sampling).

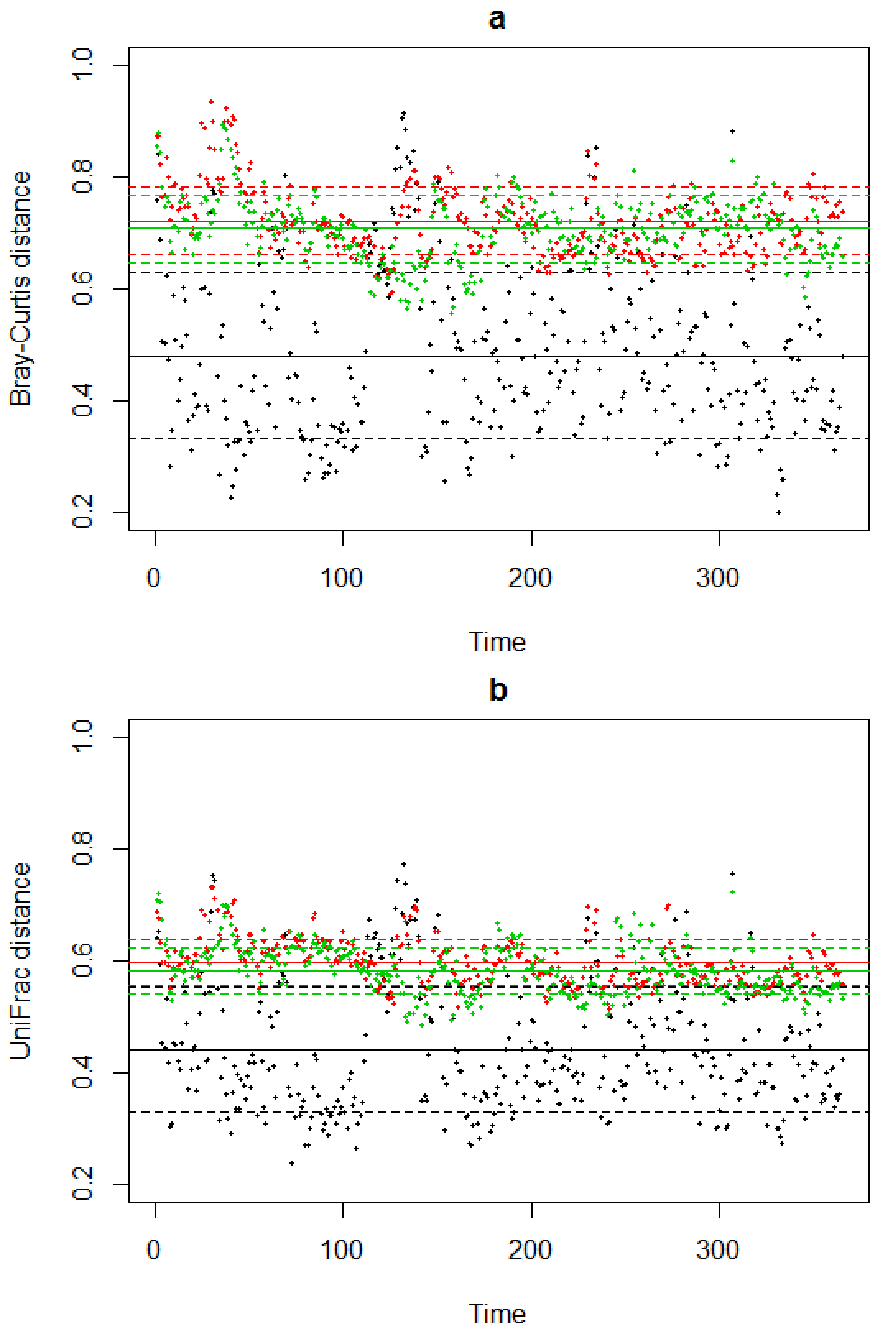

Supplementary Figure 29 | Contemporaneous distances between twins and between twins and other infants.

a. Bray-Curtis distances based on interpolated data series. The black dots are the per time point distances between the two twins (ID10 and ID11). The red dots are mean distances between ID10 and the other infants (excluding ID11 and ID8*). The green dots are mean distances between ID11 and the other infants (excluding ID10 and ID8*). The solid lines are the mean distances and the dotted lines are ±1 standard deviation. **b.** Unweighted UniFrac distances based on interpolated data series. In both **a** and **b,** the mean distance between the twins was significantly smaller than the mean distance between either twin and the other infants ($p<0.001$, t-test). The interpretation of dots and lines is as above. *ID8 was excluded from this analysis since sampling did not commence until day 54.

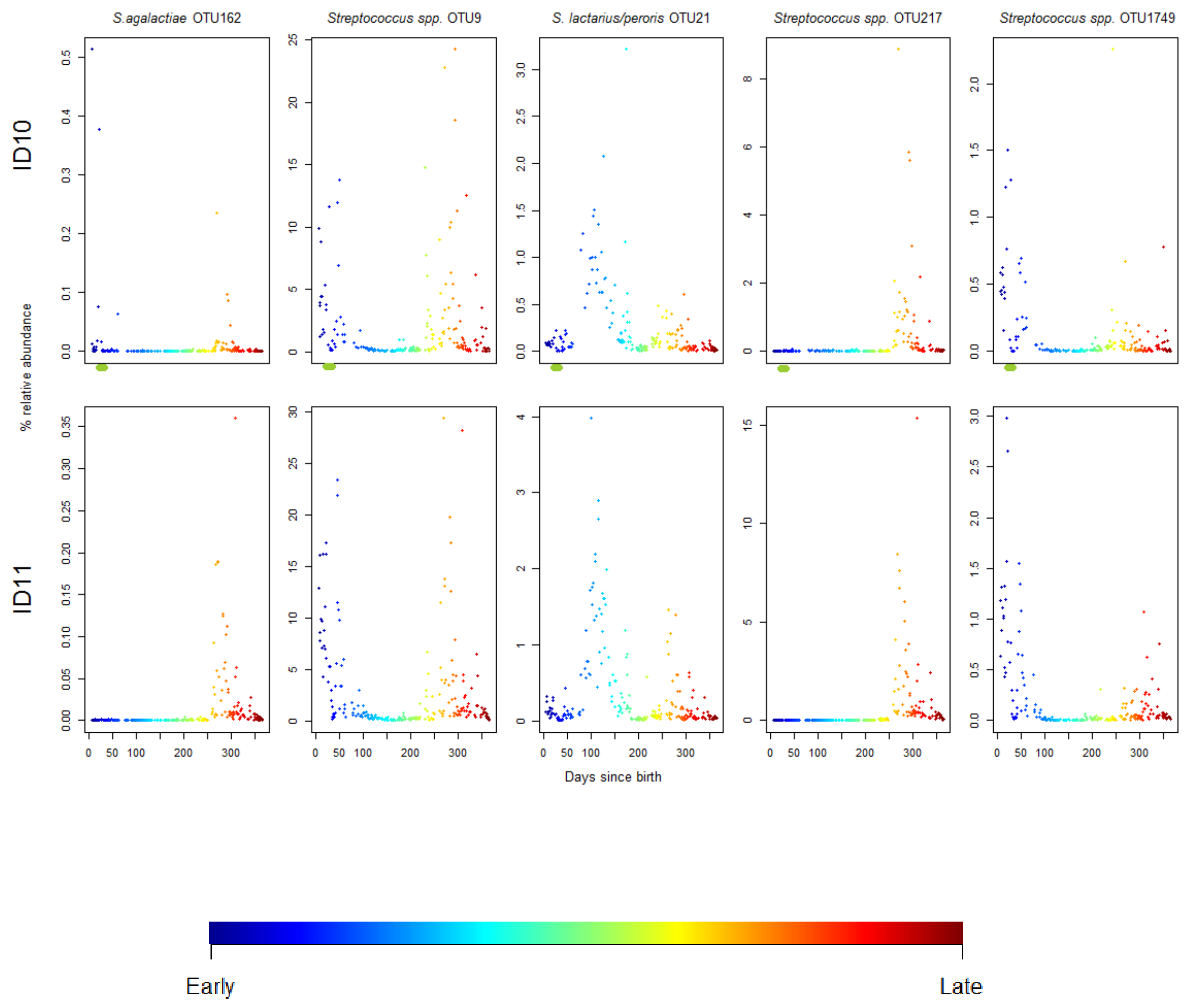

Supplementary Figure 30 | Five most abundant OTUs classified as Streptococcus. The dots show relative abundances of the OTUs. Classification is indicated above the boxes. The green bars below the top five panels indicate the period during which ID10 received antibiotics treatment. Dots are coloured according to the bar beneath the panels (see Figure 1). The relative abundance of these Streptococcus OTUs tracked each other closely in the twins despite antibiotic treatment in ID10.

Supplementary Table 1. Top twenty most abundant OTUs and their correlation with Bray-Curtis distances during the periond of convergence.

#	OTU	Mean relative abundance	Spearman correlation*	P-value*	Genus level classification	Classification confidence	Top BLAST hit of consensus sequence	Percent identity
1	OTU1	15.0 %	-0.77	<0.001	*Bifidobacterium*	0.99	*B. longum/breve*	100
2	OTU2	14.4 %	-0.43	<0.001	*Bacteroides*	0.99	*B. vulgatus*	100
3	OTU3	10.3 %	-0.39	<0.001	*Bacteroides*	1	*B. fragilis*	100
4	OTU4	6.6 %	-0.36	0.002	*Escherichia/Shigella*	0.85	multiple species	
5	OTU8	4.6 %	-0.08	NS	*Veillonella*	0.99	*V. dispar*	100
6	OTU6	4.4 %	0.5	<0.001	*Klebsiella*	0.78	multiple species	100
7	OTU5	3.8 %	0.34	0.003	*Clostridium_sensu_stricto*	0.64	*C. saccharobutylicum*	98
8	OTU7	3.4 %	-0.46	<0.001	*Lachnospiracea incerta cedis*	0.97	*Ruminococcus gnavus*	100
9	OTU9	2.4 %	0.03	NS	*Streptococcus*	1	multiple species	
10	OTU10	1.9 %	0.46	<0.001	*Clostridium_XVIII*	1	*Erysipelatoclostridium ramosum*	100
11	OTU11	1.9 %	-0.05	NS	*Haemophilus*	0.82	*H. parainfluenzae*	100
12	OTU14	1.4 %	0.74	<0.001	*Bacteroides*	0.99	*B. caccae*	100
13	OTU18	1.4 %	-0.33	0.005	*Clostridium sensu strictu*	0.98	*C. saudiense/disporicum*	100
14	OTU15	1.3 %	-0.18	NS	*Bacteroides*	0.99	*B. uniformis*	100
15	OTU13	1.2 %	0.11	NS	*Clostridium XIVa*	0.64	multiple species	
16	OTU141	1.1 %	0.31	0.007	*Clostridium sensu strictu*	0.99	*C. butyricum*	99
17	OTU12	1.1 %	-0.02	NS	*Faecalibacterium*	0.98	*F. Prausnitzii*	99
18	OTU17	1.1%	-0.24	0.04	*Bacteroides*	0.99	*B. stercoris*	100
19	OTU27	1.1 %	0.38	0.001	*Clostridium XI*	0.97	*Intestinibacter bartletti*	100
20	OTU21	1.0 %	0.15	NS	*Streptococcus*	1	*S. lactarius/peroris*	100 %
	Total	78.3 %						

*The correlation coefficients and P-values come from testing the relationship between the indicatied OTUs and the mean contemporraneous Bray-Curtis distances between the infants during the convergence period (See Figure 2).
