# Supplementary material for: Individuality and convergence of the infant gut microbiota during the first year of life
Source: Nat Commun. 2018 Jun 8;9:2233. doi: 10.1038/s41467-018-04641-7 (PMC5993781; doi:10.1038/s41467-018-04641-7)
Supplement: Supplementary file 3 — Description of Additional Supplementary Files [file 41467_2018_4641_MOESM3_ESM.pdf]

Description of Additional Supplementary Files

File Name: Supplementary Data 1
Description: OTU taxonomic classification.
